# Tyrosine Phosphoproteomics of Patient-Derived Xenografts Reveals Ephrin Type-B Receptor 4 Tyrosine Kinase as a Therapeutic Target in Pancreatic Cancer

**DOI:** 10.3390/cancers13143404

**Published:** 2021-07-07

**Authors:** Santosh Renuse, Vijay S. Madamsetty, Dong-Gi Mun, Anil K. Madugundu, Smrita Singh, Savita Udainiya, Kiran K. Mangalaparthi, Min-Sik Kim, Ren Liu, S. Ram Kumar, Valery Krasnoperov, Mark Truty, Rondell P. Graham, Parkash S. Gill, Debabrata Mukhopadhyay, Akhilesh Pandey

**Affiliations:** 1 Department of Laboratory Medicine and Pathology, Mayo Clinic, Rochester, MN 55905, USA; renuse.santosh@mayo.edu (S.R.); mun.dong-gi@mayo.edu (D.-G.M.); madugundu.anil@mayo.edu (A.K.M.); arina26@gmail.com (S.S.); savybadal@gmail.com (S.U.); mangalaparthi.kiran@mayo.edu (K.K.M.); graham.rondell@mayo.edu (R.P.G.); 2 Center for Individualized Medicine, Mayo Clinic, Rochester, MN 55905, USA; 3 Department of Biochemistry and Molecular Biology, Mayo Clinic College of Medicine and Science, Jacksonville, FL 32224, USA; madamsetty.vijay@mayo.edu; 4 Institute of Bioinformatics, International Technology Park, Bangalore 560066, Karnataka, India; 5 Manipal Academy of Higher Education (MAHE), Manipal 576104, Karnataka, India; 6 Center for Molecular Medicine, NIMHANS, Bangalore 560027, Karnataka, India; 7 Amrita School of Biotechnology, Amrita Vishwa Vidyapeetham University, Kollam 69025, Kerala, India; 8 McKusick-Nathans Institute of Genetic Medicine, Johns Hopkins University School of Medicine, Baltimore, MD 21205, USA; mkim@dgist.ac.kr; 9 Department of Biological Chemistry, Johns Hopkins University School of Medicine, Baltimore, MD 21205, USA; 10 Departments of Pathology and Medicine, Keck School of Medicine of the University of Southern California, Los Angeles, CA 90033, USA; liurenhope@hotmail.com (R.L.); parkashg@med.usc.edu (P.S.G.); 11 Departments of Surgery, Keck School of Medicine of the University of Southern California, Los Angeles, CA 90033, USA; ramkumar.subramanyan@med.usc.edu; 12 VasGene Therapeutics Inc., Los Angeles, CA 90033, USA; valery@vasgene.com; 13 Division of Hepatobiliary and Pancreas Surgery, Mayo Clinic, Rochester, MN 55905, USA; truty.mark@mayo.edu

**Keywords:** EphB4, receptor tyrosine kinase, targeted therapy, personalized medicine

## Abstract

**Simple Summary:**

Pancreatic cancer is one of the deadliest solid malignancies. Pancreatic ductal adenocarcinoma accounts for 90% of pancreatic cancer cases with minimal response to traditional chemotherapies. Protein tyrosine kinases have been shown to be hyperactivated in cancers and thus can serve as therapeutic targets. Patient-derived tumor xenografts (PDXs) in animal models such as mice are an appropriate resource to identify such activated kinases. PDXs models are excellent for the identification of therapeutic targets as compared to cell line models as they better reflect an in vivo environment. We identified ephrin type-B receptor 4 (EphB4) as hyperactivated in PDXs derived from pancreatic ductal adenocarcinoma.

**Abstract:**

Pancreatic ductal adenocarcinoma is a recalcitrant tumor with minimal response to conventional chemotherapeutic approaches. Oncogenic signaling by activated tyrosine kinases has been implicated in cancers resulting in activation of diverse effector signaling pathways. Thus, the discovery of aberrantly activated tyrosine kinases is of great interest in developing novel therapeutic strategies in the treatment and management of pancreatic cancer. Patient-derived tumor xenografts (PDXs) in mice serve as potentially valuable preclinical models as they maintain the histological and molecular heterogeneity of the original human tumor. Here, we employed high-resolution mass spectrometry combined with immunoaffinity purification using anti-phosphotyrosine antibodies to profile tyrosine phosphoproteome across 13 pancreatic ductal adenocarcinoma PDX models. This analysis resulted in the identification of 1199 tyrosine-phosphorylated sites mapping to 704 proteins. The mass spectrometric analysis revealed widespread and heterogeneous activation of both receptor and non-receptor tyrosine kinases. Preclinical studies confirmed ephrin type-B receptor 4 (EphB4) as a potential therapeutic target based on the efficacy of human serum albumin-conjugated soluble EphB4 in mice bearing orthotopic xenografts. Immunohistochemistry-based validation using tissue microarrays from 346 patients with PDAC showed significant expression of EphB4 in >70% of patients. In summary, we present a comprehensive landscape of tyrosine phosphoproteome with EphB4 as a promising therapeutic target in pancreatic ductal adenocarcinoma.

## 1. Introduction

Pancreatic ductal adenocarcinoma (PDAC) is the fourth most common cause of death from cancer in the USA and is predicted to be the second leading cause by the year 2030 [1,2]. The overall 5-year survival rate of PDAC patients from all stages of the disease combined is less than 10% and has not increased in the past five decades [3]. There are no effective therapies for PDAC that can cure or substantially increase survival, and only about 10%–20% of patients are considered candidates for surgery as most patients present with metastatic cancer. Gemcitabine chemotherapy in combination with the EGFR inhibitor erlotinib showed modest improvements in the overall survival rate [4]. Thus, characterization of the underlying molecular mechanisms responsible for the development of PDAC may lead to the identification of novel therapeutic targets. Several genetic alterations, including KRAS, CDKN2A, TP53, SMAD4, and BRCA2, have been implicated in the pathogenesis of PDAC [5,6].

Aberrant activation of kinase signaling pathways is commonly observed in several malignancies. Activating mutations in kinases such as EGFR represent a major mechanism of oncogenesis. Kinases regulate most cellular processes responsible for cell growth and differentiation through reversible phosphorylation. Thus, kinases have emerged as attractive candidates for targeted therapy in a number of cancers [7,8]. Phosphoproteomics offers a functional readout in biological systems reflecting proteins and pathways that are activated, thus acting as a surrogate to identify activated kinases. We and others have used this approach to characterize aberrant kinase signaling in various cancers and demonstrated its utility in identifying potential therapeutic targets [9,10,11]. Thus, the identification of oncogenic tyrosine kinases activated across a broad spectrum of PDACs is a promising strategy for the development of new targeted therapies and for individualizing treatment.

Various kinases have been found to be implicated in PDAC development and progression, such as EGFR, which is required for acinar-ductal metaplasia (ADM). Overexpression of EGFR and related receptor, ERBB3, has been detected in PDAC [12], whereas pseudokinase 223 (SgK223) has been shown to be increased by phosphorylation in PDAC cell line models through JAK1/Stat3 signaling [13]. Tao et al. studied proteome and phosphoproteome of patient-derived PDAC tissues and identified several extracellular matrix-related proteins associated with PDAC [14]. Le Large et al. studied the phosphoproteome of 11 pancreatic cancer cell lines and identified focal adhesion kinase (FAK) as a potential synergistic target with Nab-paclitaxel [15]. Humphrey et al. carried out phosphotyrosine profiling of 36 PDAC cell lines and observed three different patterns based on the profiles [16]. However, there is still a lack of studies describing phosphotyrosine signaling in PDAC tissues and PDX models.

In this study, we carried out phosphotyrosine profiling of a number of PDXs to discover activated tyrosine kinase signaling pathways in PDAC. We employed antibody-based enrichment of phosphotyrosine peptides followed by a liquid chromatography-mass spectrometry-based label-free quantitation approach to identify and quantify phosphotyrosine peptides among these PDXs. In all, we identified 1199 unique phosphorylation sites mapped to 704 proteins in one or more PDX samples. While there was considerable heterogeneity in the phosphopeptides identified across individual cases, we identified several recurring activated tyrosine kinases such as the EPH receptor B4 (EphB4). We validated EphB4 overexpression using pancreatic ductal adenocarcinoma tissue microarrays (TMAs) using immunohistochemistry (IHC). In vivo studies directed against EphB4 using albumin-conjugated soluble EphB4 in combination with gemcitabine showed a significant decrease in tumor growth in orthotropic xenografts. Overall, our results show that EphB4 could serve as a therapeutic target in the treatment of pancreatic ductal adenocarcinoma.

## 2. Materials and Methods

### 2.1. Reagents

Anti-phosphotyrosine antibody (HRP conjugated, 4G10) was purchased from Millipore (Billerica, MA, USA). Anti-phosphotyrosine mouse monoclonal antibody (pY1000) coupled to beads for immunoaffinity purification of tyrosine-phosphorylated peptides was purchased from Cell Signaling Technology (Danvers, MA, USA). TPCK-treated trypsin was from Worthington Biochemical Corp. (Lakewood, NJ, USA). HPLC-grade water and LC-MS grade acetonitrile were from ThermoFisher Scientific (San Jose, CA, USA).

### 2.2. Establishment of PDX Model

Establishing an appropriate preclinical animal model is crucial for translational cancer research. One popular method that has been widely adopted is grafting cancer cell lines derived from patients. We followed the same protocol (i.e., homogenizing the patient tissues and using the cell lines that were generated) to develop orthotopic PDX tumor models. As we used cell lines derived from patients’ tissues, the success rate was >80%. For orthotopic pancreatic cancer animal models, 6- to 8-week-old NSG male mice were procured from Charles River Laboratories and housed in the institutional animal facilities. All animal experiments were approved by the Institutional Animal Care and Use Committee of Mayo Clinic. To establish an orthotopic pancreatic tumor model, PDAC tumor cells were suspended in 100 mL of PBS containing 20% matrigel were slowly injected orthotopically into the head pancreas. Post-tumor growth, the mice were sacrificed, and PDXs were resected for phosphoproteomics experiments.

### 2.3. Processing of Patient-Derived Xenografts for Peptide Preparation

PDX tissues were pulverized in liquid nitrogen. The pulverized samples were lysed in urea lysis buffer (20 mM HEPES pH 8.0, 9 M urea, 1 mM sodium orthovanadate, 2.5 mM sodium pyrophosphate, and 1 mM ß-glycerophosphate) and sonicated for 30 ss twice on ice. Cell lysates were cleared by centrifugation at 20,000× *g* at 15 °C for 15 min. Protein samples of 25 mg from each were reduced with 5 mM dithiothreitol for 60 min at 37 °C and alkylated with 10 mM iodoacetamide for 30 min in the dark at room temperature. Samples were diluted with 20 mM HEPES pH 8.0 to a final concentration of 2 M urea and incubated with TPCK-treated trypsin at room temperature overnight with gentle end-to-end shaking. Protein digests were acidified by adding 20% trifluoroacetic acid (TFA) to a final concentration of 1% TFA and subjected to centrifugation at 2000× *g* at room temperature for 15 min. The supernatant of protein digests was loaded onto a Sep-Pak C_18_ cartridge (Waters Corporation, Milford, MA, USA) pre-equilibrated with 0.1% TFA. Peptides were eluted with 40% acetonitrile with 0.1% TFA. Eluted peptides were lyophilized and subjected to immunoaffinity purification of tyrosine-phosphorylated peptides.

### 2.4. Immunoaffinity Purification of Tyrosine Phosphopeptides

Immunoaffinity purification (IAP) of tyrosine-phosphorylated peptides was carried out as previously described [11]. Briefly, after lyophilization, about 25 mg of tryptic peptides was dissolved in 1.4 mL of 1× IAP buffer (50 mM MOPS pH 7.2, 10 mM sodium phosphate, 50 mM NaCl) and subjected to centrifugation at 2000× *g* at room temperature for 5 min. Before immunoaffinity purification, P-Tyr-1000 beads were washed with 1× IAP buffer twice at 4 °C, and the pH of the supernatant containing peptides was adjusted to ~7.2 by adding 1 M Tris Base. For immunoaffinity purification, the supernatant was incubated with P-Tyr-1000 beads at 4 °C for 60 min, and the beads were washed three times with 1× IAP buffer and twice with cold ultrahigh pure water. Peptides captured by antibody were eluted twice by incubating the beads with 0.15% TFA at room temperature for 15 min. Eluted peptides were desalted by C_18_ STAGE tip, vacuum dried, and kept at −80 °C prior to LC-MS/MS experiments.

### 2.5. Liquid Chromatography-Tandem Mass Spectrometry

LC-MS/MS analysis of the enriched tyrosine-phosphorylated peptides was carried out using a reversed-phase liquid chromatography system (Ultimate 3000 RSLCnano, Thermo Scientific, San Jose, CA, USA) connected online to an Orbitrap Fusion Lumos mass spectrometer (Thermo Scientific, San Jose, CA, USA). The peptides were loaded onto a trap column (PepMap C_18_ 2 cm × 100 µm, and 100 Å) at a flow rate of 20 µL/min using 0.1% formic acid and separated on an analytical column (Aurora 25 cm × 75 µm, C_18_ 1.6 µm, and 100 Å, IonOpticks, Victoria, Australia) with a flow rate of 300 nL/min with a linear gradient of 5% to 30% solvent B (100% acetonitrile, 0.1% formic acid) over a 120 min. Both precursor and fragment ions were acquired in the Orbitrap mass analyzer. Precursor ions were acquired in *m*/*z* range of 350–1800 with a resolution of 120,000 at 200 *m*/*z*. Precursor fragmentation was carried out using the higher-energy collisional dissociation method using a normalized collision energy of 27. The fragment ions were acquired at a resolution of 30,000 at 200 *m*/*z*. The scans were arranged with the top-speed method with 3 s of cycle time between MS and MS/MS. Ion transfer capillary voltage was maintained at 2.3 kV. For internal mass calibration, the lock mass option was enabled with polysiloxane ion (*m*/*z*, 445.120025) from ambient air [17].

### 2.6. Mass Spectrometry Data Analysis

The acquired mass spectra were searched using Andromeda in MaxQuant [18] software suite (version 1.5.1.3) against a UniProt human protein database, including common MS contaminants. The search parameters included a maximum of two missed cleavages; carbamidomethylation at cysteine as a fixed modification; N-terminal acetylation, oxidation at methionine, phosphorylation at serine, threonine, and tyrosine as variable modifications. The precursor tolerance was set to 10 ppm and MS/MS tolerance to ±0.02 Da. The false discovery rate was set to 1% at the peptide-spectrum matches (PSMs), peptide, and protein level. Label-free quantitation (LFQ) was used for the quantitation of peptides based on precursor ion abundance. The phosphorylation site localization probability for each Ser/Thr/Tyr site was calculated and filtered using a 75% cut-off.

### 2.7. Hierarchical Clustering

Hierarchical Clustering Explorer (version 3.5, http://www.cs.umd.edu (accessed on 23 February 2021)) was used to create tyrosine phosphorylation-based clustering using tyrosine-phosphorylated peptides. Normalized/averaged spectral abundances were loaded onto the software. When clustering, the Pearson correlation distance metric and average linkages were set. A color gradient was used, with black being non-detected and red being the highest expression.

### 2.8. Western Blotting

PDXs were pulverized in liquid N2 and the resulting fine tissue powder was lysed in modified RIPA buffer (10 mM Tris HCl, pH 7.4, 100 mM NaCl, 1 mM EDTA, 1 mM EGTA, 1 mM NaF, 0.5% sodium deoxycholate, 1% Triton X-100, 10% glycerol, 0.1% SDS and 1 mM sodium orthovanadate in the presence of protease inhibitors). Equal amounts of lysates from PDX samples were separated on Bolt™ 4%–12% Bis-Tris Plus Gels (Invitrogen, Grand Island, NY, USA). Proteins were transferred to nitrocellulose membrane and probed with anti-EphB4 antibody (Cell Signaling Technology, Danvers, MA, USA). The lysates were subjected to immunoprecipitation of phosphotyrosine phosphorylated proteins using agarose-conjugated pan anti-phosphotyrosine antibody (EMD Millipore, Burlington, MA, USA) and probed with HRP-conjugated anti-phosphotyrosine antibody (EMD Millipore, Burlington, MA, USA) and/or anti-EphB4 antibody (Cell Signaling Technology, Danvers, MA, USA).

### 2.9. In Vivo sEphB4 Treatment

A total of 2 × 10^6^ CAPAN-1 cells were orthotopically implanted into the pancreas of 10–12 weeks male athymic mice to establish tumors. On day 20, mice were randomized to 4 groups and treated by intraperitoneal injection of PBS, sEphB4-HSA (20 mg/kg, thrice a week), gemcitabine (10 mg/kg, twice a week), and a combination of sEphB4-HSA and Gemcitabine. On day 30, mice were examined for abdominal girth and jaundice. On day 40, the mice were scanned with PET-CT. On day 100, mice were sacrificed, and liver, lung, and pancreas were harvested for both macroscopic pathological examination and Western blot analysis.

### 2.10. Immunohistochemical Labeling

Validation of EphB4 was performed on a large number of samples (*n* = 346) using tissue microarrays (TMAs). Eighteen TMAs included treatment naïve adenocarcinoma samples consisting of 346 unique individuals with adenocarcinoma. Each TMA consisted of 60 tissue cores of 2.0 mm diameter. Up to three cores per subject, along with control tissue from 28 individuals with other pancreatic conditions (pancreatitis, serous cystadenoma, and normal pancreas) cores were placed on the TMA using a random layout. These slides were baked overnight at 65 °C prior to deparaffinization. The tissue sections were deparaffinized in xylene (2 × 10 min) followed by absolute alcohol (5 min) and 95% alcohol (5 min). Following this, the sections were transferred to a 3% *v*/*v* solution of hydrogen peroxide in methanol for 20 min for blocking endogenous peroxidases. The sections were then transferred to 70% alcohol (2 min) followed by 0.05 M Tris-buffered saline (TBS), pH 7.6. Antigen retrieval was carried out using citrate buffer (10 mM Citric Acid, 0.05% Tween 20, pH 6.0) in a pressure cooker for 20 min. The slides were allowed to cool down to room temperature. The slides were transferred to TBS. A solution of 2.5% normal horse serum (Vector Laboratories, Inc., Burlingame, CA, USA) was applied to the tissue sections for 30 min to block endogenous biotin. The primary antibody anti-EphB4 (Cell Signaling Technology, Danvers, MA, USA) was applied to the slides at a dilution of 1:100 and incubated for 2 h at room temperature. The slides were washed in TBS (2 changes, 5 min each). A horseradish peroxidase-conjugated anti-mouse/anti-rabbit polyclonal IgG antibody was used as the secondary antibody (Vector Laboratories, Inc., Burlingame, CA, USA). The slides were incubated with the secondary antibody for 30 min and then washed in TBS (2 changes, 5 min each). The slides were then incubated for 5 min in a 1% solution of 3,3′-diaminobenzidine peroxidase substrate. The slides were washed in distilled water (two changes, 2 min each), counterstained with Harris hematoxylin for 30 ss, and washed in running tap water for 2 min. Positive and negative external controls were included in each staining run. Dehydration and clearing were done by incubating the slides in sequential order in 95% alcohol for 2 min and absolute alcohol (2 changes for 3 min each). The clearing was done in xylene (2 changes, 5 min each). Sections were mounted using DPX and appropriate coverslips and incubated at 50 °C for 15 min for drying. The slides were examined by a Pathologist for the intensity and distribution of staining in the tumor tissue cores in all 18 TMAs. Immunostaining was evaluated and scored using the semi-quantitative H-score method to calculate the sum of the percentage and intensity of positively stained tumor cells within the invasive tissue component. The H-score ranged from 0 to 300, where H-score = (1 × (% cells 1+) + 2 × (% cells 2+) + 3 × (% cells 3+)). Each tumor specimen was scored once, where three FFPE cores representing the same tumor from the same patient were averaged. A tumor sample with an H score ≥ 50 was considered positive.

## 3. Results

### 3.1. Phosphotyrosine Profiling in PDX Models of Pancreatic Ductal Adenocarcinoma

**Figure 1:**
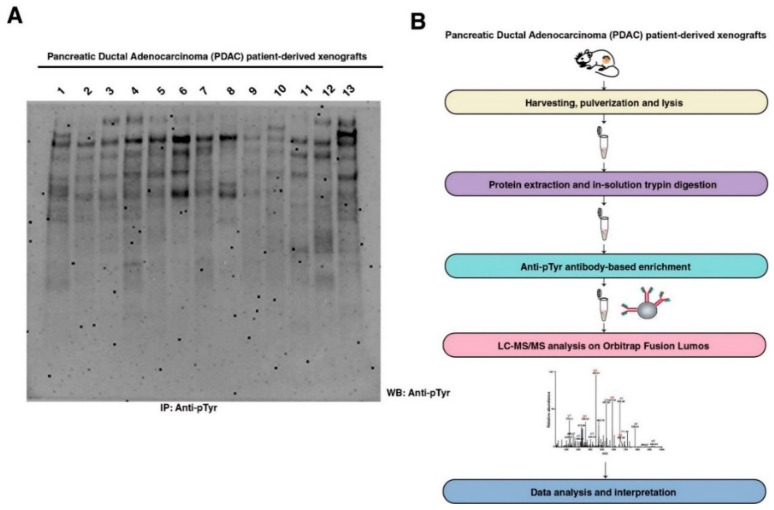
Phosphotyrosine profiling of pancreatic ductal adenocarcinoma (PDAC) patient-derived xenografts (PDXs): (**A**) Global phosphotyrosine characterization of PDAC PDX samples using immunoprecipitation and Western blot with anti-phosphotyrosine antibody (Clone 4G10); (**B**) Thirteen PDXs were pulverized in liquid nitrogen followed by protein extraction using 9 M urea lysis buffer in 20 mM HEPES (pH 7.4) containing phosphatase inhibitors. Twenty-five mg protein from each PDX sample was subjected to in-solution trypsin digestion followed by SepPak C_18_ clean-up and lyophilization. Anti-phosphotyrosine antibody was used for immunoaffinity purification of phosphotyrosine peptides, followed by LC-MS/MS analysis.

**Figure 2:**
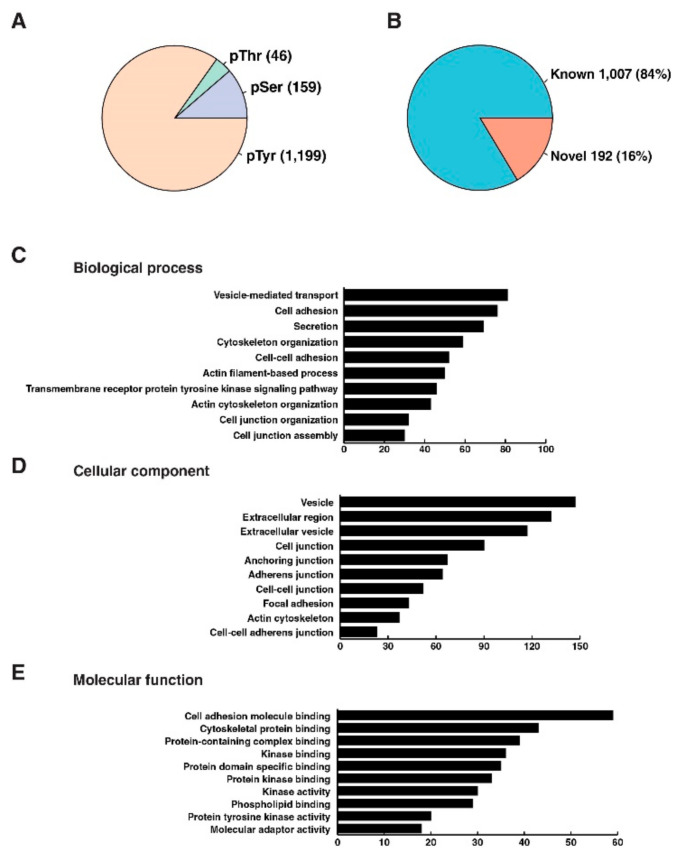
Summary of tyrosine phosphoproteome and gene ontology analysis: (**A**) Phosphosites identified from humans included 1199 phosphotyrosine, 159 phosphoserines, and 46 phosphothreonine with unambiguous site localization; (**B**) 192 of the identified phosphosites constituted 16% of identified phosphosites were found to be novel based on PhosphoSitePlus database; (**C**–**E**) Ingenuity pathway analysis (IPA) was used for gene ontology analysis including biological process (**C**), cellular component (**D**) and molecular function (**E**). The x-axes indicate the number of proteins in each classification.

There is a remarkable genetic diversity in pancreatic cancer, as indicated by recent exome sequencing studies on PDAC, not only between primary and metastatic cancer but also within distinct metastatic lesions [19,20,21]. Thus, it is anticipated that tyrosine kinome would be heterogeneously activated as well and was confirmed by Western blot analysis of global tyrosine phosphorylation in a panel of 13 PDXs (Figure 1A). As observed in Figure 1A, the tyrosine phosphorylation pattern of each individual PDX sample is quite distinct. To characterize the global tyrosine phosphorylation status in pancreatic ductal adenocarcinoma PDXs, we employed an immunoaffinity profiling of phosphotyrosine peptides as described previously [10]. Briefly, PDX sample lysates were digested using trypsin, and phosphotyrosine peptides were enriched using an anti-phosphotyrosine antibody. The enriched phosphopeptides were analyzed by LC-MS/MS, and label-free quantitation was carried out as outlined in Figure 1B. We identified 1199 unique tyrosine phosphorylation sites at least in one PDX sample corresponding to 704 proteins (Figure 2A). A total of 192 phosphotyrosine sites were found to be novel based on community curated data deposited in PhosphoSitePlus [22] (Figure 2B). A total of 23 tyrosine phosphosites (20 proteins) were identified from the 13 PDX samples. This included kinases such as EGFR, MAPK13, CDK1, LYN, EPHA2, FRK, and PTK6. We had previously identified EGFR as well as EPHA2 being hyperphosphorylated in pancreatic cancer and esophageal cancer, respectively [11,12]. A total of 391 phosphotyrosine sites were identified, mapping to 259 proteins in at least five PDX samples.

### 3.2. Activated Tyrosine Kinome

To understand the potential functional implications of phosphorylation in these PDX samples, we analyzed gene ontology (GO) enrichment of phosphoproteins identified in at least five PDX samples. GO analysis showed that phosphorylation was enriched for proteins that are involved in vesicle-mediated transport, cell adhesion, secretion, and transmembrane receptor tyrosine kinase signaling pathway in biological processes. With respect to cellular components, phosphorylation was mainly detected in proteins associated with cell junction, focal adhesion, extracellular region, cell-cell adherence junction, while phosphorylation of proteins involved in molecular functions such as cell adhesion molecule binding, cytoskeletal protein binding, kinase activity, and protein tyrosine kinase activity was found to be enriched (Figure 2C–E). Several receptor tyrosine kinases such as EPHB4, EPHA2, EPHB3, EGFR, ERBB3, and DDR1 were found to be phosphorylated. In addition, domain analysis using the SMART tool showed enrichment of several important regulatory modules in cellular signaling cascades such as src homology 2, Src homology 3, tyrosine kinase catalytic domain, and pleckstrin homology domains implying that intracellular signal networks are highly regulated by these tyrosine-phosphorylated proteins. These results again confirm that our analysis focusing on tyrosine phosphoproteome specifically enriched signaling molecules involved in early events in signal transduction pathways.

**Figure 3:**
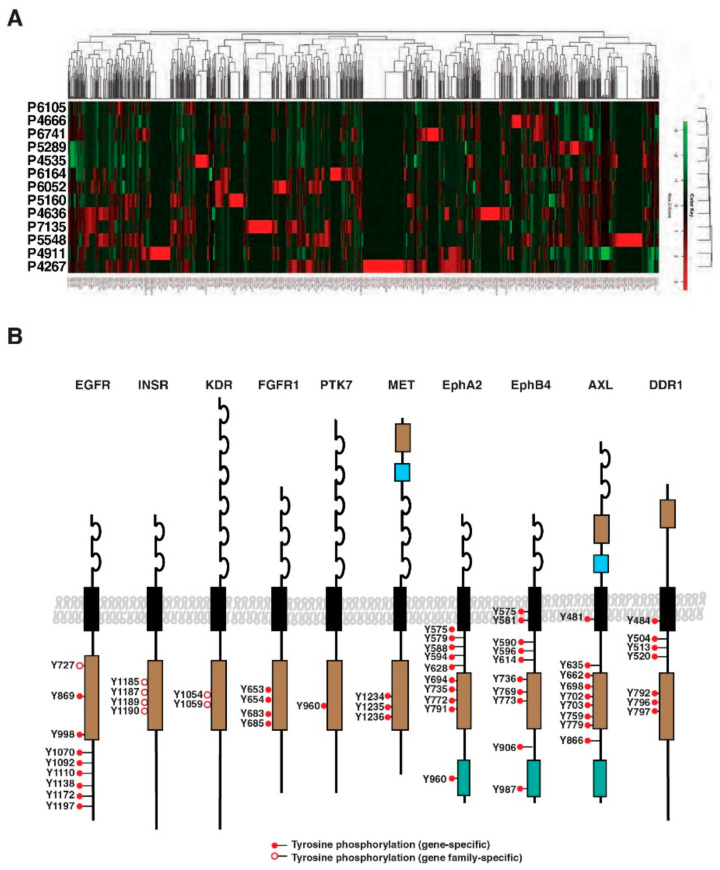
Widespread tyrosine activation in PDAC PDXs: (**A**) Unsupervised hierarchical clustering shows the distinct pattern of tyrosine phosphorylation in PDXs used in this study from humans. PDX sample numbers are indicated on the left margin. The color key shows peptide intensities from low (green) to high (red); (**B**) Several receptor tyrosine kinase families were found to be hyperphosphorylated, including EGFR and Ephrin receptors. All the tyrosine kinase domains were found to be tyrosine-phosphorylated, while juxta-membrane domains were phosphorylated only in some families, such as the Ephrin receptor and DDR.

Unsupervised hierarchical clustering analysis was carried out to determine if there was a pattern of activated tyrosine kinases or tyrosine kinase subfamilies. As shown in Figure 3A, a diverse pattern of tyrosine phosphorylation was observed across all PDX samples recapitulating the heterogeneous nature observed by genetic analysis. We observed that many receptor tyrosine kinases and non-receptor tyrosine kinases were hyperphosphorylated, although the level of phosphorylation varied across individual PDX samples. Tyrosine phosphorylation sites identified in all 13 PDX samples are listed in Supplementary Table S1.

Reversible protein tyrosine phosphorylation is mediated by protein tyrosine kinases and phosphatases whose deregulation has been implicated in many cancers. Autophosphorylation at tyrosine residues of receptor tyrosine kinases within activation loops and juxtamembrane regions prompted aggressive proliferative signal transduction [23]. Among subfamilies of tyrosine kinases previously reported as activated in PDAC, several hyperphosphorylated examples were identified, including receptor tyrosine kinases (RTKs) such as EGFR, ERBB3, and MET, as shown in Figure 3B [12,16,24]. Receptor tyrosine kinases were found to be phosphorylated in their activation loops within kinase domains, and ephrin receptor tyrosine kinases have hyperphosphorylation events at their juxtamembrane regions (Figure 3B). A complete list of RTKs and non-RTKs in which increased tyrosine phosphorylation was observed is shown in Supplementary Table S1. As previously reported by our group [12], downstream molecules activated by EGFR were also phosphorylated, validating the functional status of the EGFR signaling pathway. Similarly, we have recently reported that the Axl receptor tyrosine kinase is differentially activated in metastatic PDAC and is correlated with the burden of the metastases [25]. In addition, we discovered many RTKs that have previously not been reported to be activated in PDAC, including some of the members of the Eph receptor subfamily.

### 3.3. Eph Receptor Tyrosine Kinases Hyperphosphorylated in PDAC PDXs

**Figure 4:**
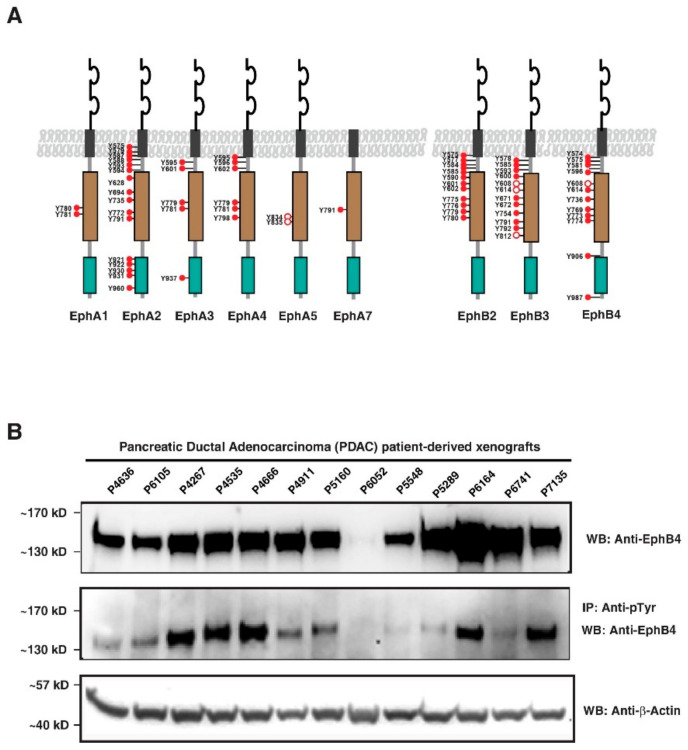
Highly activated ephrin receptors: (**A**) As indicated in red dots, most of the ephrin receptor tyrosine kinases were found to be tyrosine phosphorylated with EphA2 and EphB4 being most hyperphosphorylated; (**B**) Western blot showing total EphB4 expression (top panel), phosphorylated EphB4 (middle panel) and actin as a loading control (bottom panel) in PDX samples. Full pictures of the Western blots are shown in Supplementary Materials.

In our screen of tyrosine phosphoproteome in PDX samples, we identified members of the Eph receptor subfamily to be activated on the number of tyrosine residues. As shown in Figure 4A, Eph receptor subfamily proteins such as EphA1, EphA2, EphA4, EphA5, EphA7, EphB2, EphB3, and EphB4 were identified to be phosphorylated at various tyrosine residues. Eph receptor tyrosine kinases and their ephrin ligands are involved in bidirectional signaling in various signaling pathways and implicated in various tumors through critical physiological and pathological processes [26,27]. Our group has previously shown EphA2 hyperphosphorylation in esophageal cancer based on cell line phosphotyrosine profiling data [11], whereas EphA2 and EphB4 have been upregulated in breast cancer [28]. In this study, we observed hyperphosphorylation of EphB4 on as many as nine tyrosine residues in at least 50% of PDX samples. Immunoprecipitation of tyrosine-phosphorylated proteins followed by immunoblotting using anti-EphB4 showed high expression of EphB4 in 7/13 of the PDX samples (Figure 4B—Middle panel), indicating EphB4 hyperphosphorylation in pancreatic cancer. We observed a low signal for EphB4 phosphorylation in sample P6052 by Western blotting, which was correlated with phosphosite intensities identified by mass spectrometry. On the other hand, a similar correlation was not observed for samples P5548 and P5289, where the Western blot revealed a low to undetectable signal, although the mass spectrometry phosphosite data showed moderate to high signal. The discrepancy observed could be due to different detection techniques used. The total EphB4 expression was also seen to be higher in pancreatic cancer; however, relatively lower expression in the normal pancreas [29]. Public data repositories such as GTEx (https://www.gtexportal.org/home/gene/EPHB4 (accessed on 2 June 2021)) and the Human Protein Atlas (https://www.proteinatlas.org/ENSG00000196411-EPHB4/tissue/primary+data (accessed on 2 June 2021)) show lower expression of EphB4 in the pancreas but a relatively higher expression in pancreatic cancer.

The study by Lennon et al. indicated a role of EphB4-EphrinB2 in the treatment of resistance to radiotherapy in pancreatic ductal adenocarcinoma [29]. The study showed the involvement of the tumor microenvironment in the release of immunosuppressive factors, thereby contributing to tumor progression, and targeted therapies in combination with radiotherapy could arrest the tumor growth.

### 3.4. EphB4 as a Novel Therapeutic Target in Pancreatic Cancer

**Figure 5:**
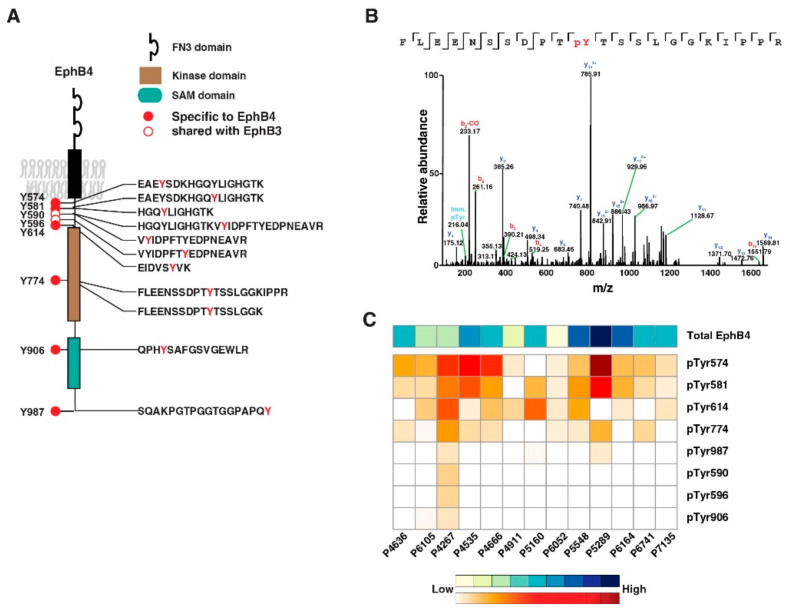
EphB4 receptor tyrosine kinase was hyperphosphorylated: (**A**) Localization of eight tyrosine phosphosites detected, including two that are shared with EphB3 receptor tyrosine kinases; (**B**) Representative tandem MS/MS spectrum of tyrosine-phosphorylated peptide (FLEENSSDPTpYTSSLGGKIPPR). The presence of y12^+^ and y12^2+^ ions provides evidence for phosphorylation on Y774 of EphB4 receptor tyrosine kinase; (**C**) Abundance of EphB4 was found to be correlated with the tyrosine phosphorylation levels measured by phosphotyrosine profiling using LC-MS/MS analysis across PDX samples. Total EphB4 levels are shown in the top row of the heatmap, while individual phosphosites are shown below as indicated. The color key indicates low to high expression.

**Figure 6:**
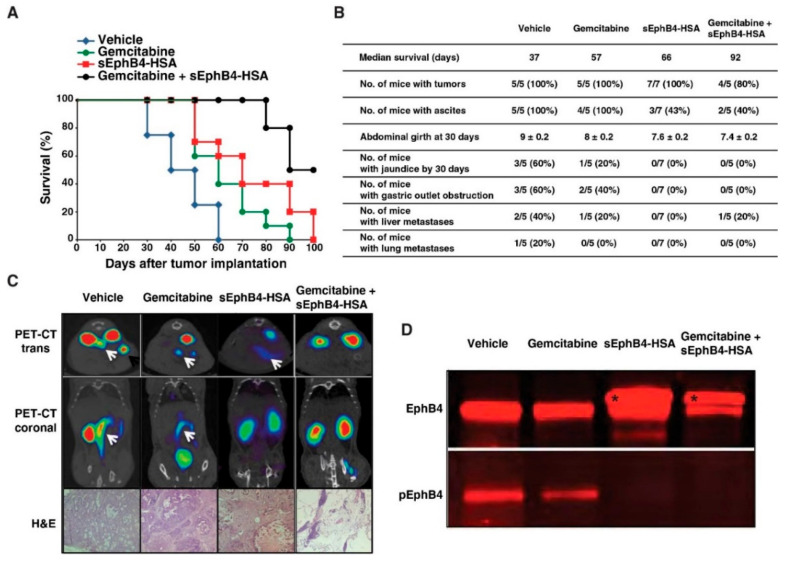
sEphB4 inhibits tumor growth and metastasis in an orthotopic tumor model. CAPAN-1 cells were orthotopically implanted into the pancreas of athymic mice to establish tumors. Mice were randomized into four groups and treated by intraperitoneal injection of PBS, sEphB4-HSA (20 mg/kg, thrice a week), gemcitabine (10 mg/kg, twice a week), and a combination of sEphB4-HSA and gemcitabine: (**A**) The survival results of the study (terminated at day 100); (**B**) Pathological analysis summary of the mice at the end of the study; (**C**) Representative PET-CT scan pictures. Tumors are indicated with white arrows. Representative H&E staining pictures of the pancreas are shown at the bottom; (**D**) Expression of EphB4 and its phosphorylation. EphB4 phosphorylation was inhibited by sEphB4-HSA treatment in the tumor as compared to vehicle-treated samples.

In our study, we observed hyperphosphorylation of EphB4 among other Eph receptor tyrosine kinases. As shown in Figure 5A, eight distinct phosphotyrosine sites were identified from EphB4. Figure 5B shows representative MS/MS spectrum FLEENSSDPTpYTSSLGGKIPPR indicates the identification of Y774 phosphosite on EphB4. As shown in Figure 5C, we also observed that EphB4 overexpression in most of the PDX tumor models suggesting the involvement of activated EphB4 in pancreatic ductal adenocarcinoma. Highly activated EphB4 in PDAC can serve as a therapeutic target. The binding of the EphB4 receptor to its cognate ligand EphrinB2 leads to receptor and ligand dimerization, which in turn leads to autophosphorylation of the intracellular domain of EphB4 and activation of downstream signaling. We thus synthesized a soluble monomeric form of the extracellular domain of EphB4 [30]. This protein binds EphrinB2, prevents normal EphB4-EphrinB2 interaction and their subsequent phosphorylation [30,31]. In addition, sEphB4-HSA that has human serum albumin fused to EphB4′s C-terminus to prolong its half-life in circulation, has shown profound anti-tumor activities in preclinical models [30,32,33,34]. We thus tested sEphB4-HSA efficacy in an orthotopic PDAC model using a human CAPAN-1 cell line. Established CAPAN-1 tumors were treated with sEphB4-HSA, gemcitabine, or sEphB4-HSA combined with gemcitabine to see if the deactivation of EphB4 could lead to increased survival in vivo. As shown in Figure 6A, sEphB4-HSA treatments also significantly extended survival time from 37 days in the control group to 66 days. The pathological examination further showed sEphB4-HSA treatment inhibited tumor metastasis, reducing the total metastasis rate from 60% in controls to 0% (Figure 6B). One of the major limitations in pancreatic cancer is suboptimal penetration of drugs into the tumor due to desmoplastic reaction in the tumor and poor vascularity [35]. sEphB4-HSA, a large protein molecule, may not have penetrated the tumor optimally, leading to the growth of the primary tumor and eventual death of mice even after the disappearance of metastases. PET-CT scan showed that sEphB4-HSA significantly inhibited tumor growth compared to control xenografts (Figure 6C). In addition, when used in combination with traditional chemotherapy gemcitabine, there was an additive effect on animal survival and complete remission in one out of five animals. Tissue analysis showed that EphB4 phosphorylation is inhibited by sEphB4-HSA treatment in the tumor (Figure 6D, Bottom panel), consistent with our in vitro results. EphB4 has a prominent role in tumor migration, invasion, and metastasis [36]. Blocking tumor metastasis is the most difficult challenge in clinical practice. sEphB4-HSA administration blocked tumor metastasis from primary pancreatic cancer. It is thus highly likely that EphB4 targeted therapy in spontaneous tumor models will prevent tumor development and progression. Overall, these results indicate that EphB4 is a promising target for pancreatic cancer treatment that needs to be explored further.

### 3.5. Expression of EphB4 in Pancreatic Ductal Adenocarcinoma

**Figure 7:**
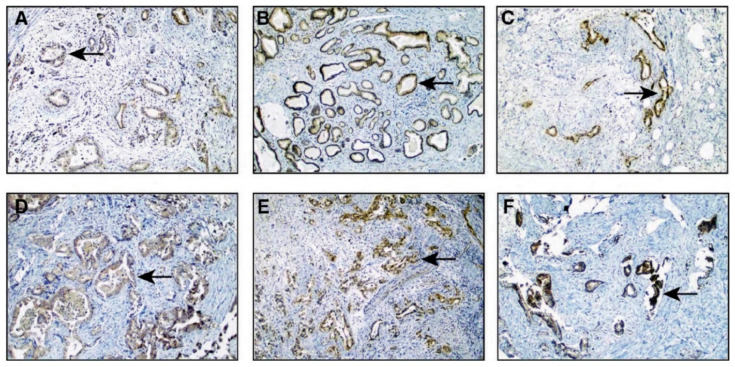
EphB4 immunostaining of pancreatic ductal adenocarcinoma. The panel shows a magnified view (200×) of infiltrating tumor regions in representative cores from tissue microarrays. The photomicrographs are ordered with increasing EphB4 expression (from (**A**–**F**)), as evidenced by the intensity of brown color development using anti-EphB4 antibody-based immunostaining shown by arrows.

To validate the overexpression of EphB4 in PDAC and determine its localization, we performed immunohistochemistry on 18 tissue microarrays (TMAs) of treatment naïve PDAC consisting of 346 unique individuals with adenocarcinoma using an anti-EphB4 antibody. Immunostaining was evaluated by a pathologist and scored using the semi-quantitative H-score method to calculate the sum of the percentage and intensity of positively stained tumor cells within the invasive tissue component [37]. Each tumor specimen was scored once, with averaging of three FFPE cores representing the same tumor from the same patient. A tumor sample with an H score ≥ 50 was considered positive. Notably, 72.3% of the tumor samples showed significant expression of EphB4 (H-score ≥ 50). Representative photomicrographs of EphB4 expression in tumor cells of PDAC specimens are shown in Figure 7.

## 4. Discussion

In this study, we used a high-resolution mass spectrometry-based label-free quantitative tyrosine phosphoproteomics approach to comprehensively profile PDAC patient-derived xenografts (PDXs) tyrosine phosphorylation. We generated an expansive view of tyrosine kinase activity and downstream signaling networks. We found 1199 phosphotyrosine sites on 704 proteins. We observed diverse activation of receptors as well as non-receptor tyrosine kinases across these samples. We were able to identify certain highly recurrent phosphorylation events that were also shown to have a functional effect on tumorigenesis. In particular, we demonstrated that EphB4 is highly activated in pancreatic cancer PDXs and is amenable to targeting with different agents, including antibodies and small-molecule kinase inhibitors. We also showed increased cytoplasmic expression of EphB4 in tumor cells in treatment naïve tumor tissue from PDAC cases by IHC, which further supports our findings. PDAC classification could be important for patient stratification and management for effective treatment. In this regard, Humphrey et al. carried out phosphotyrosine profiling of PDAC cell lines and described three different subtypes based on distinct tyrosine phosphorylation profiles (signaling networks associated with cell-cell adhesion and epithelial-mesenchyme transition, mRNA metabolism, and receptor tyrosine kinase (RTK) signaling) [16].

Ephrin receptors are activated by their specific ligands called ephrins, leading to the activation of signaling pathways [38]. Based on their structures and sequences, ephrins are divided into the ephrin-A class, which are anchored to the membrane by a glycosylphosphatidylinositol linkage, and the ephrin-B class, which are transmembrane proteins. The Eph family of receptors is divided into two groups based on the sequence similarity of their extracellular domains and their affinities for binding ephrin-A or ephrin-B ligands. The signaling pathway downstream of the receptor is referred to as forwarding signaling, while the signaling pathway downstream of the ephrin ligand is referred to as reverse signaling [38]. EphB4, together with its cognate ligand/functional ligand ephrin-B2, plays a central role in heart morphogenesis and angiogenesis through the regulation of cell adhesion and cell migration. EphB4-mediated forward signaling controls cellular repulsion and segregation from ephrin-B2-expressing cells. This interaction also plays a role in postnatal blood vessel remodeling, morphogenesis and permeability and is thus important in the context of tumor angiogenesis. EphB4 is overexpressed in many epithelial cancers [39,40,41]. It promotes tumor cell proliferation, migration, and survival [33,39]. EphB4 expressing on the tumor cells also interacts with ephrinB2 expressed on tumor vasculature to promote tumor angiogenesis [30]. Thus, hyperphosphorylation of EphB4 may be essential to the oncogenic potential of PDAC. We performed proof-of-concept in vivo experiments using orthotopic PDAC xenografts in mice and showed that neutralization EphB4-ephrinB2 interaction inhibited tumor growth and metastasis, suggesting that EphB4 is a relevant and druggable therapeutic target for this disease. This large-scale profiling of activated tyrosine kinases in a panel of PDAC PDXs provides a dataset for developing tools for prognosis, develop targeted novel therapeutics, and potentially an early measure of response. However, the exact molecular mechanisms downstream of EphB4 activation leading to tumor cell proliferation and metastasis are not known, and further studies are required to investigate the underlying pathways and protein-protein interactions.

EphB4 is known to be expressed in the vasculature in mouse embryos but not in adult animals except for the tumor vasculature [42]. EphB4 knockouts in mice are embryonically lethal, but its deletion in adult mice has no phenotype making it a very attractive therapeutic target in terms of toxicity. For example, EGFR targeted antibody causes skin toxicity, and HER2 targeted therapy causes cardiac toxicity because of the distribution of the targets in these anatomical sites. We believe that EphB4 activation could be more directly studied in the future using antibodies or multiple reaction monitoring (MRM) mass spectrometry-based approaches. Overall, the unbiased phosphoproteome in pancreatic cancer has provided us with a large set of data to further characterize novel targets for testing their biological role, correlate activation with genetic alterations, and develop multiplexed assays assessing activated tyrosine kinases in an effort to tailor therapy according to individual patient profiles.

## 5. Conclusions

This study constitutes of high-resolution mass spectrometry in combination with anti-phosphotyrosine antibody-based enrichment of tyrosine-phosphorylated peptides from 13 pancreatic ductal adenocarcinoma PDXs. We observed widespread and heterogeneous activation of tyrosine kinases. Several Eph receptor kinase family members were found to be hyperphosphorylated. EphB4 expression was observed in >70% patients by immunohistochemistry on tissue microarrays consisting of 346 patients. Based on in vivo mice experiments using human serum albumin-conjugated soluble EphB4, it was confirmed that EphB4 could serve as a potential therapeutic target in pancreatic ductal adenocarcinoma and further research is necessary to prove its efficacy in the treatment of pancreatic cancer patients.

## Data Availability

Raw mass spectrometry files used in this experiment have been uploaded to the ProteomeXchange Consortium (http://proteomecentral.proteomexchange.org, via the PRIDE partner repository [43] with the dataset identifier PXD021729.

## Supplementary Materials

The following are available online at https://www.mdpi.com/article/10.3390/cancers13143404/s1, Table S1: List of phosphotyrosine sites and proteins identified, Full pictures of the Western blots are shown in supplementary materials.
