# Supplementary figures and images for: Tyrosine Phosphoproteomics of Patient-Derived Xenografts Reveals Ephrin Type-B Receptor 4 Tyrosine Kinase as a Therapeutic Target in Pancreatic Cancer
Source: Cancers (Basel). 2021 Jul 7;13(14):3404. doi: 10.3390/cancers13143404 (PMC8303779; doi:10.3390/cancers13143404)

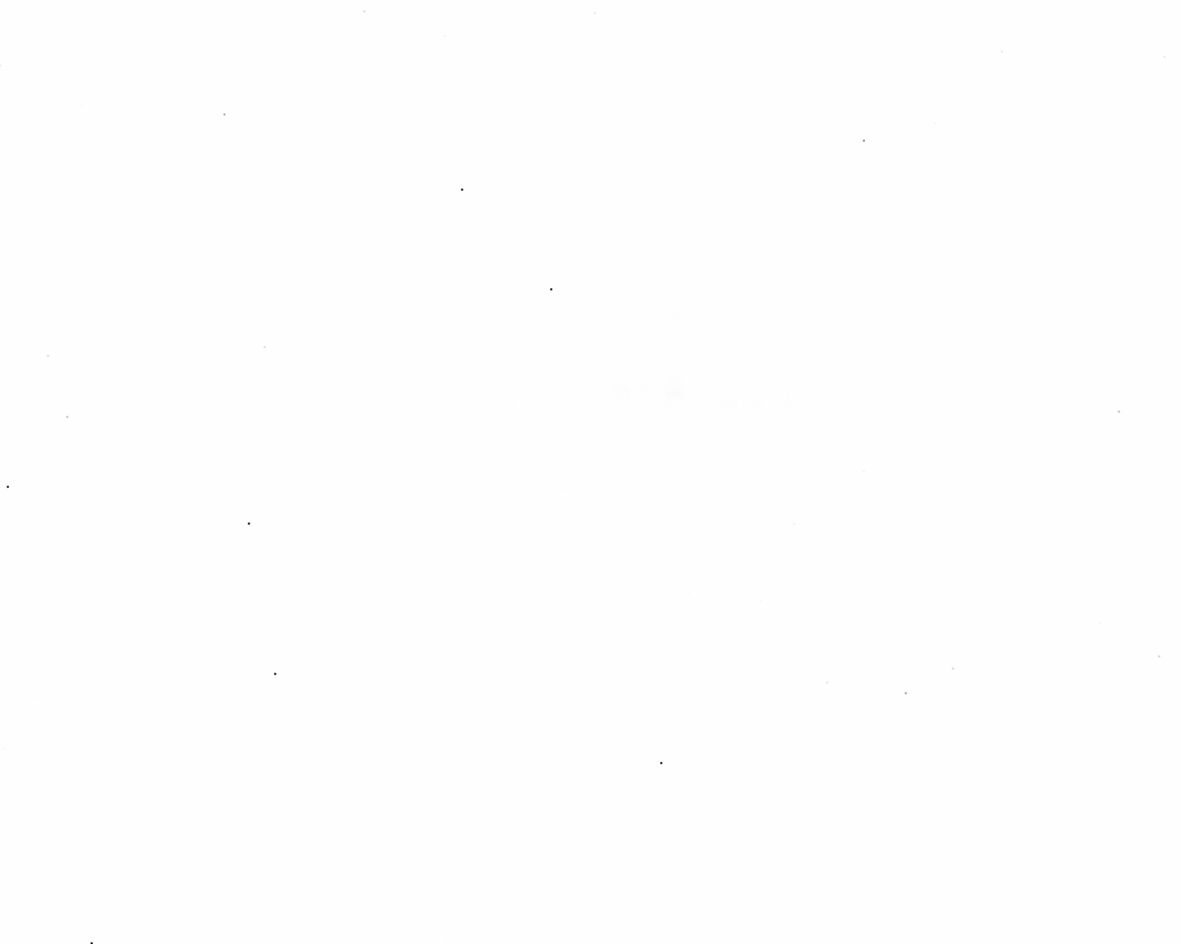

Supplement: Supplementary file 1 [file cancers-13-03404-s001.zip › CHEMI_03022020_180655.jpg]

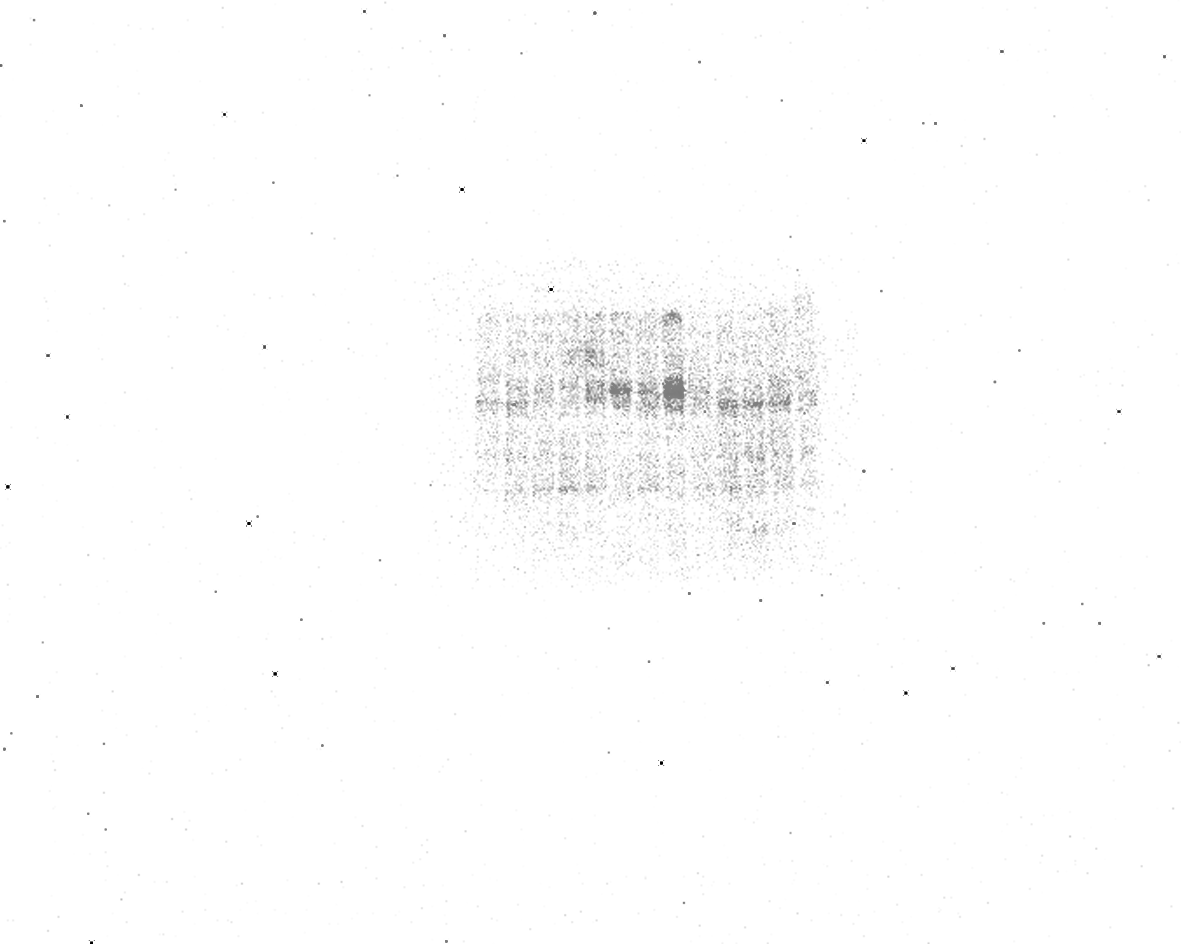

Supplement: Supplementary file 1 [file cancers-13-03404-s001.zip › CHEMI_03022020_180655.tif]

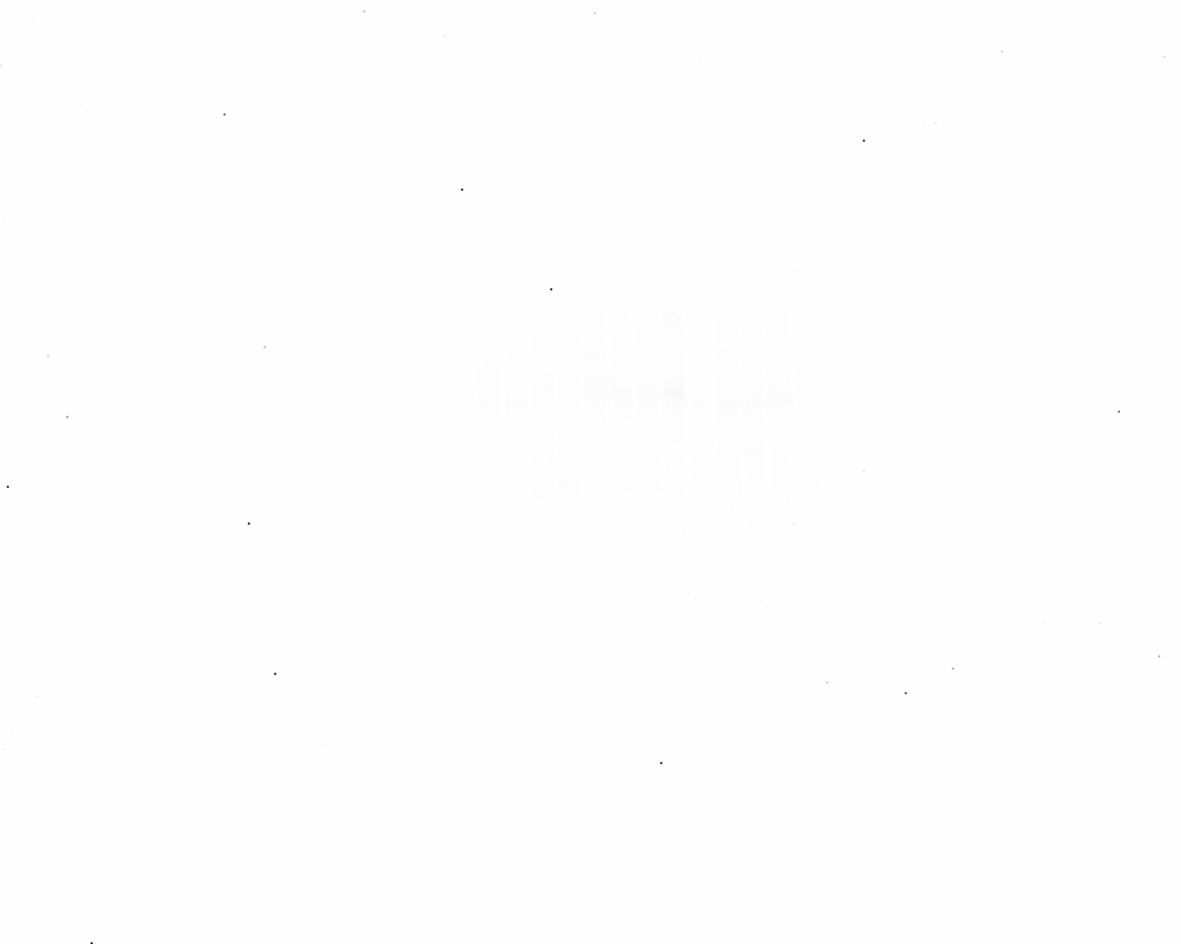

Supplement: Supplementary file 1 [file cancers-13-03404-s001.zip › CHEMI_03022020_180733.jpg]

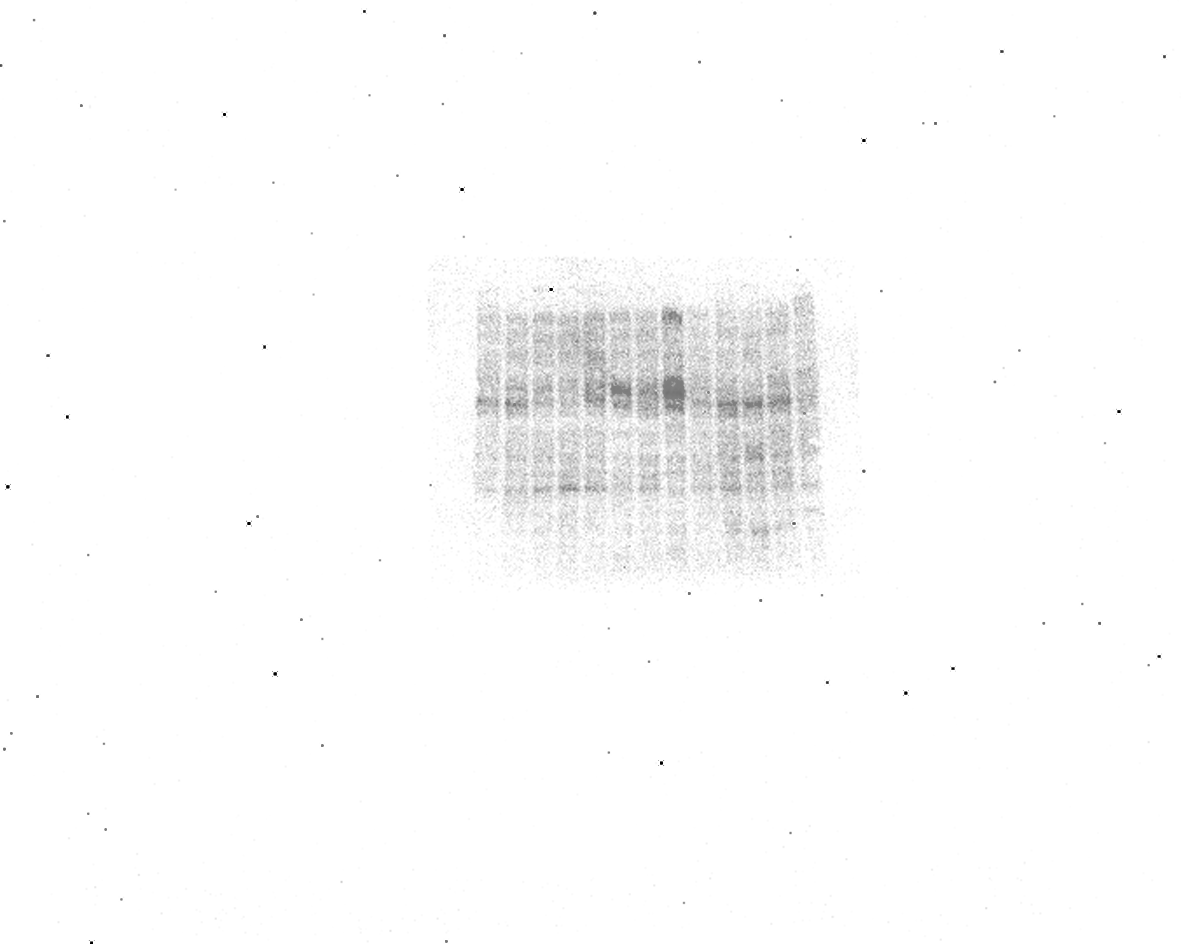

Supplement: Supplementary file 1 [file cancers-13-03404-s001.zip › CHEMI_03022020_180733.tif]

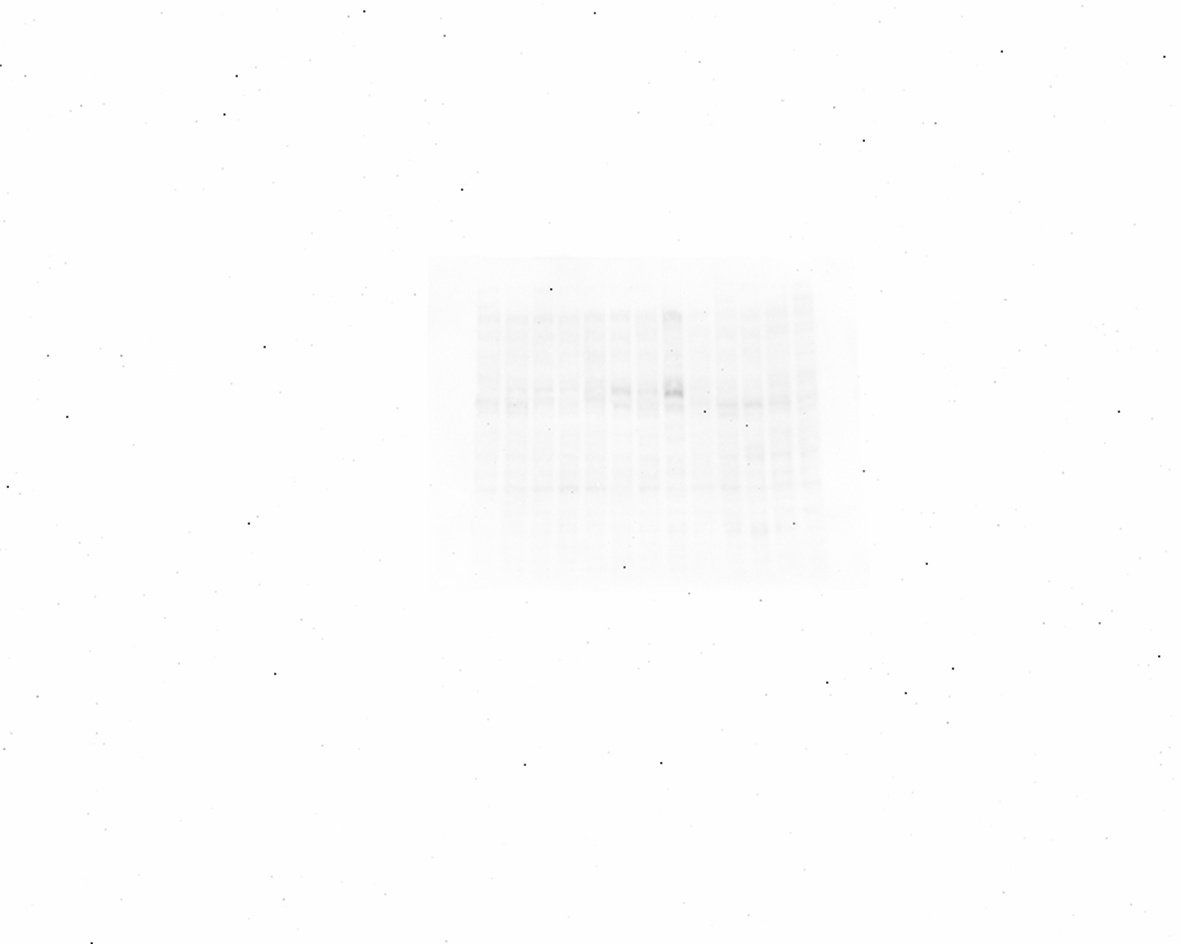

Supplement: Supplementary file 1 [file cancers-13-03404-s001.zip › CHEMI_03022020_180843.jpg]

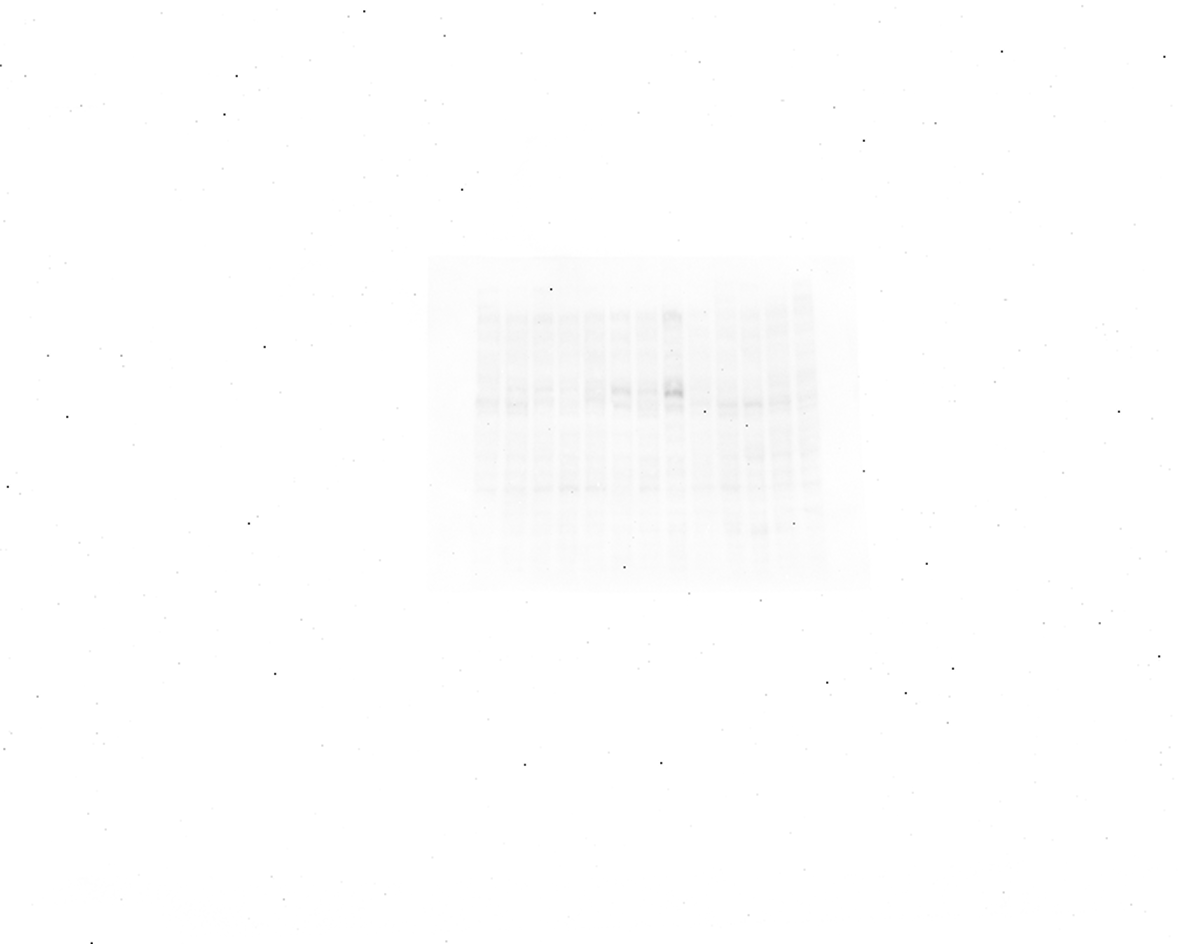

Supplement: Supplementary file 1 [file cancers-13-03404-s001.zip › CHEMI_03022020_180843.tif]

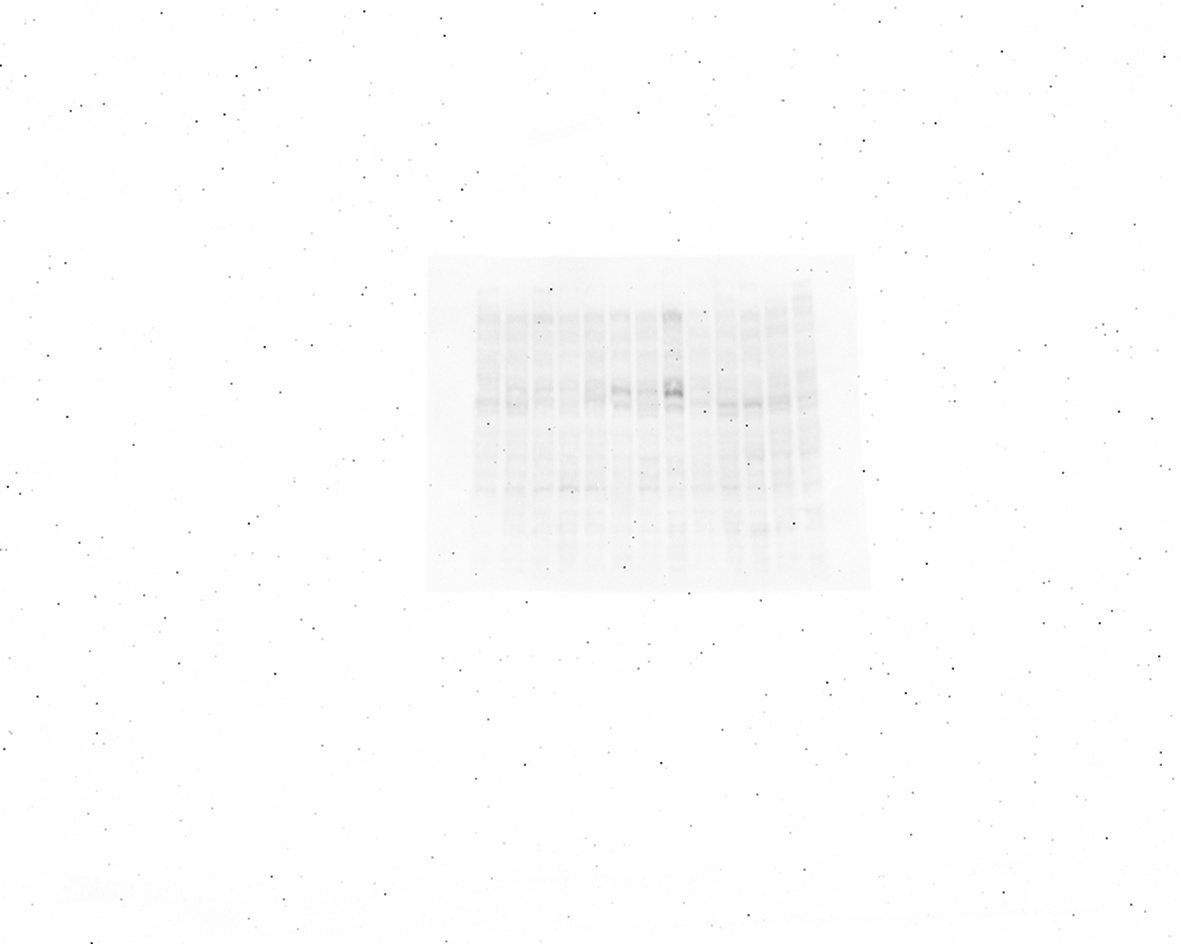

Supplement: Supplementary file 1 [file cancers-13-03404-s001.zip › CHEMI_03022020_181353.jpg]

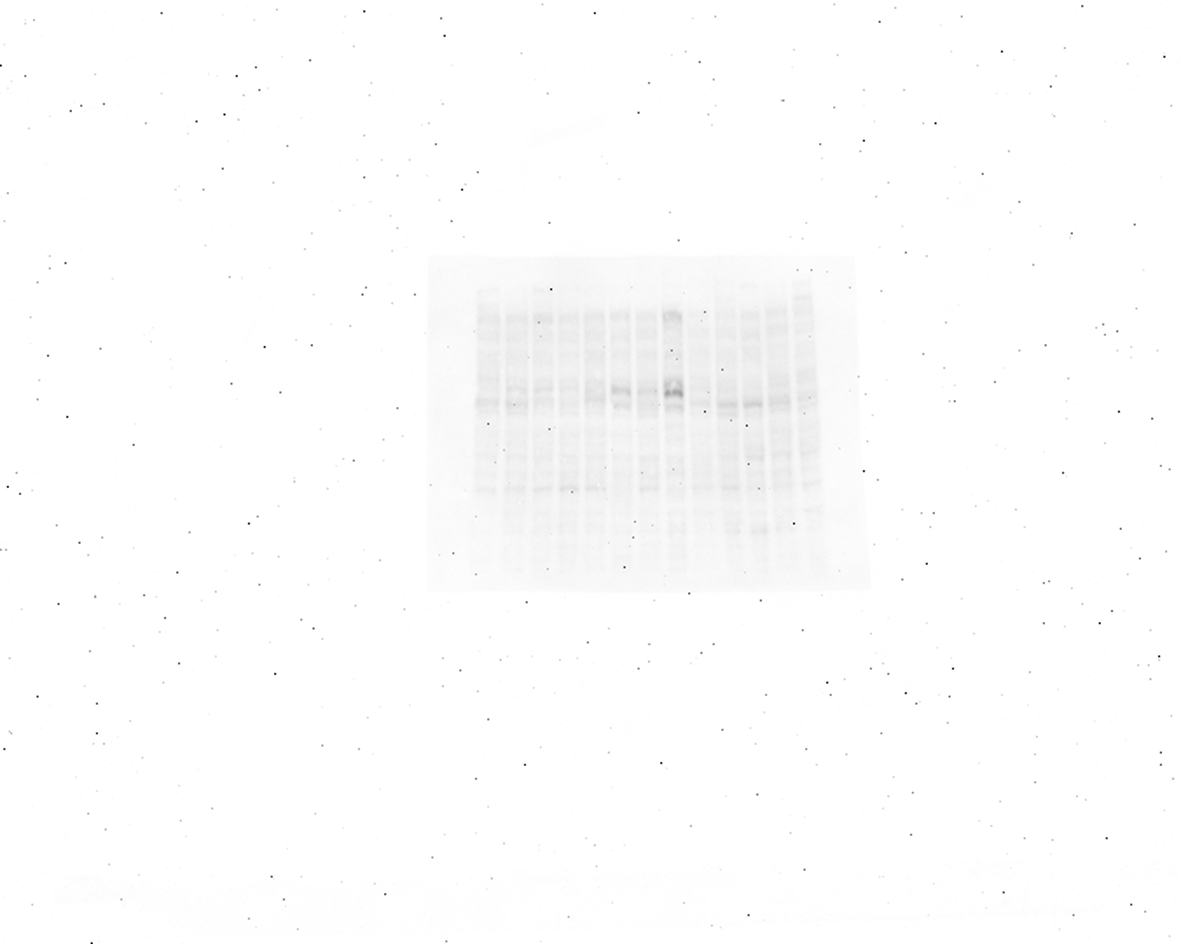

Supplement: Supplementary file 1 [file cancers-13-03404-s001.zip › CHEMI_03022020_181353.tif]

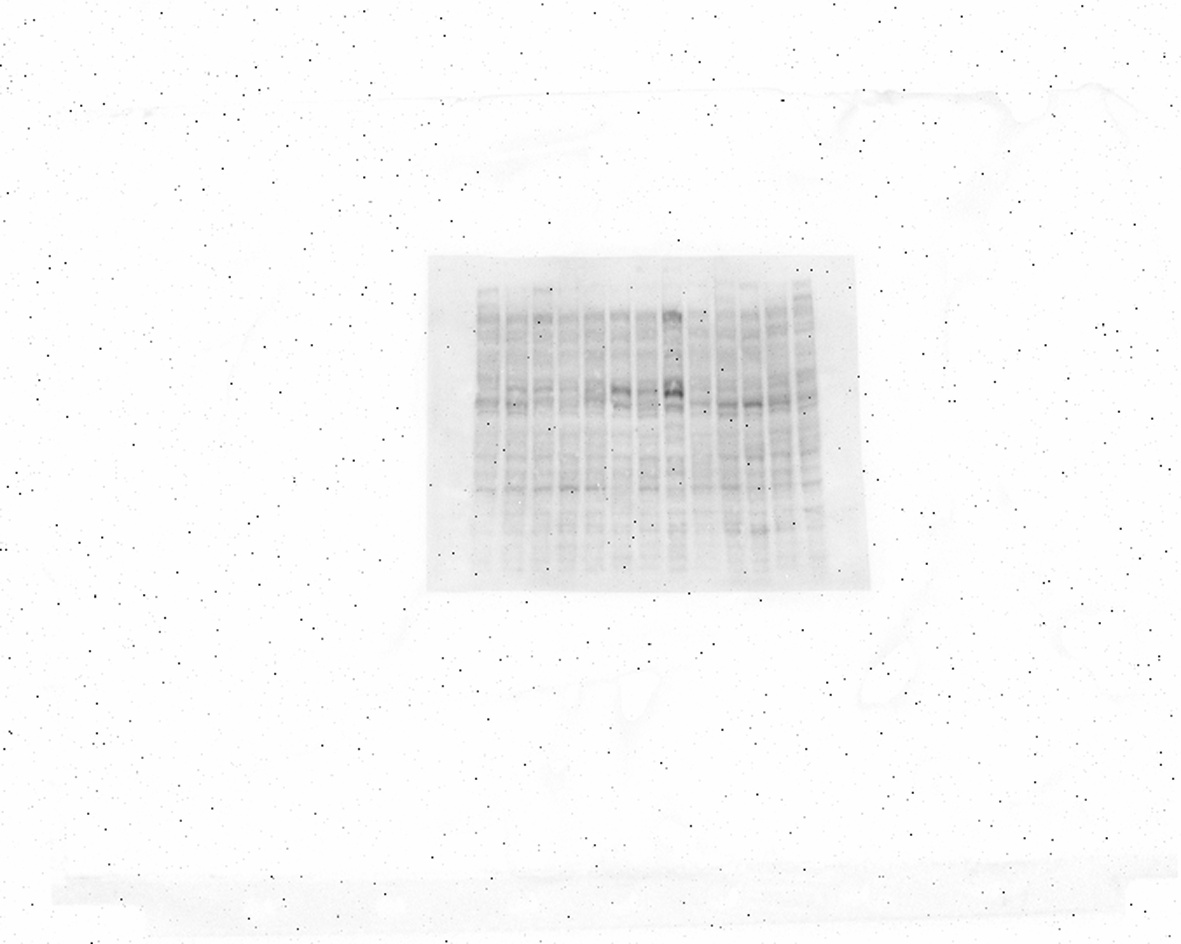

Supplement: Supplementary file 1 [file cancers-13-03404-s001.zip › CHEMI_03022020_182402.jpg]

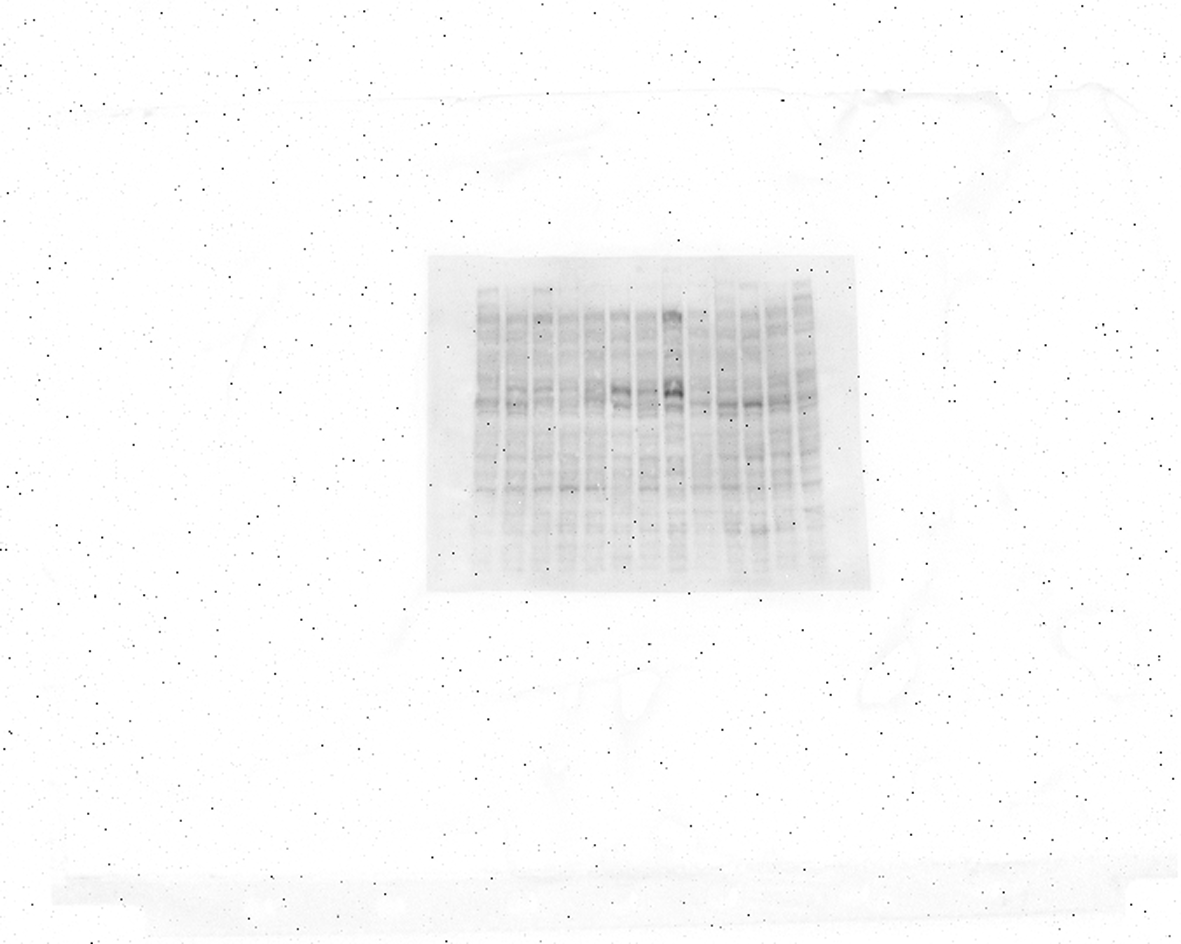

Supplement: Supplementary file 1 [file cancers-13-03404-s001.zip › CHEMI_03022020_182402.tif]

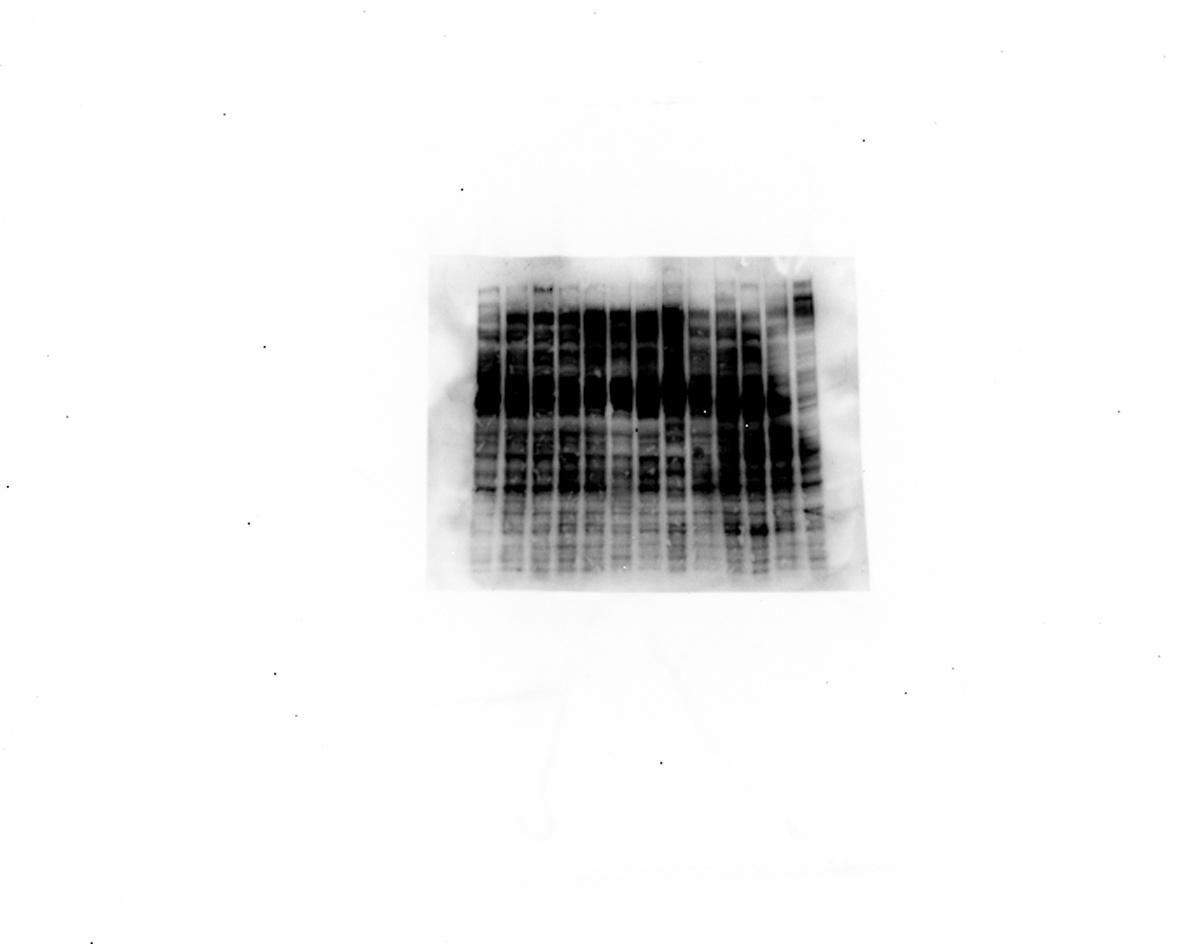

Supplement: Supplementary file 1 [file cancers-13-03404-s001.zip › CHEMI_03022020_185234.jpg]

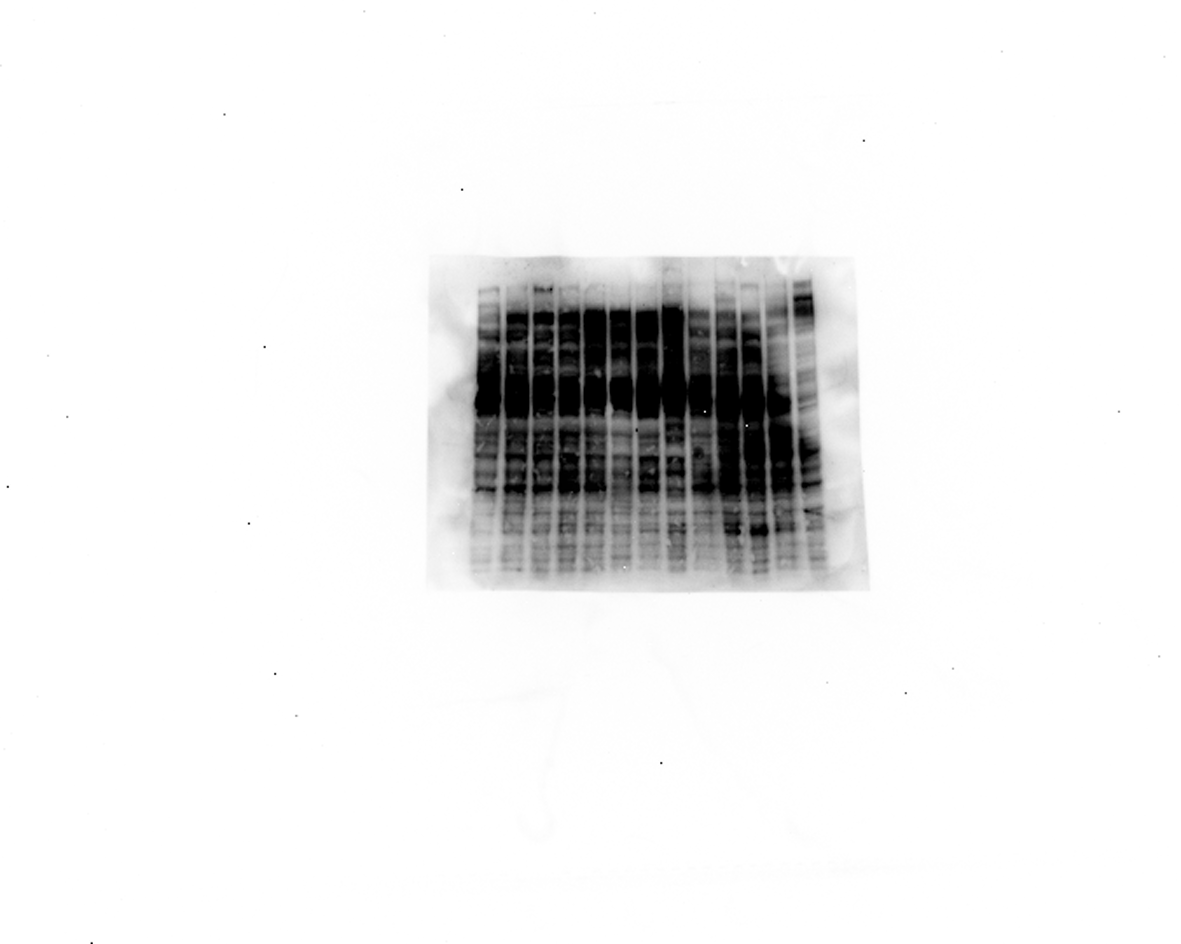

Supplement: Supplementary file 1 [file cancers-13-03404-s001.zip › CHEMI_03022020_185234.tif]

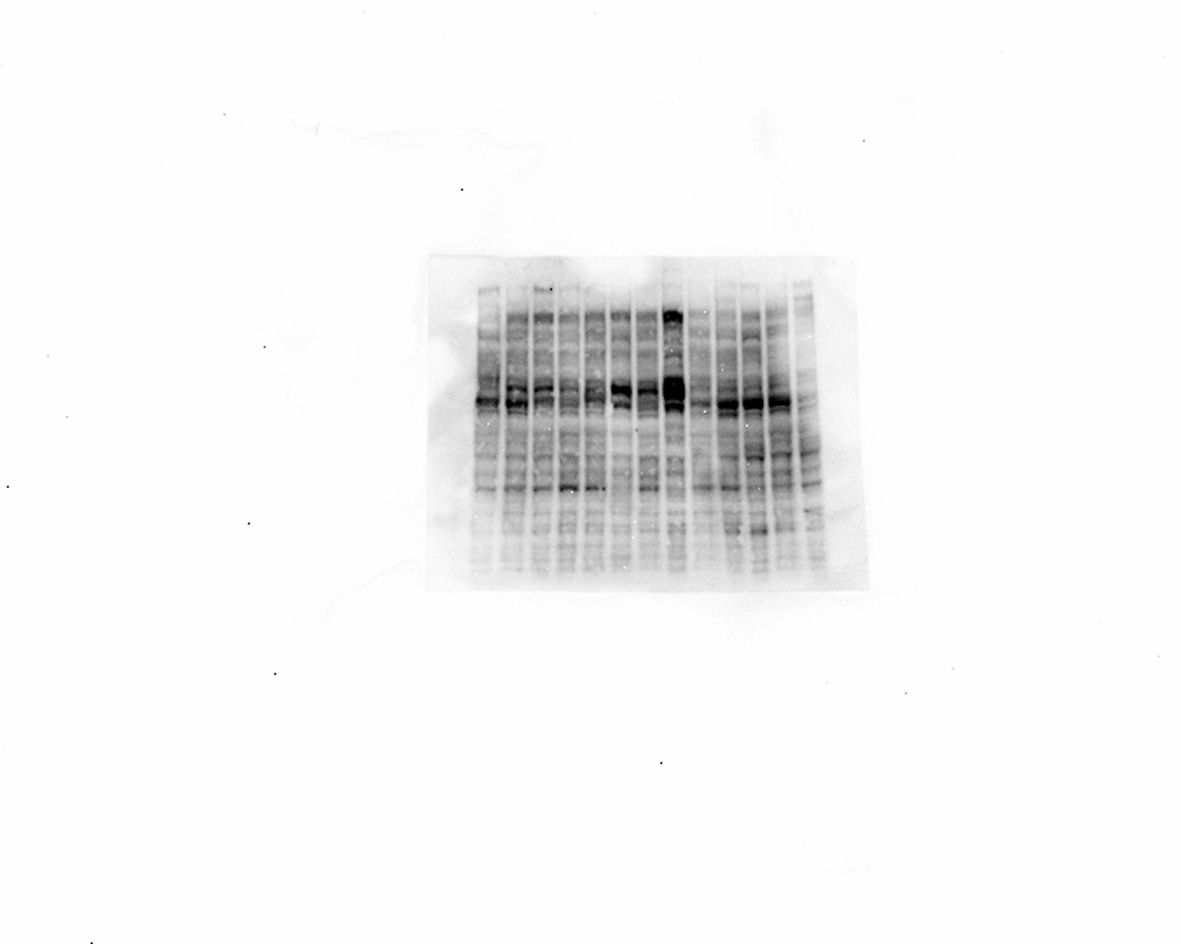

Supplement: Supplementary file 1 [file cancers-13-03404-s001.zip › CHEMI_03022020_185549.jpg]

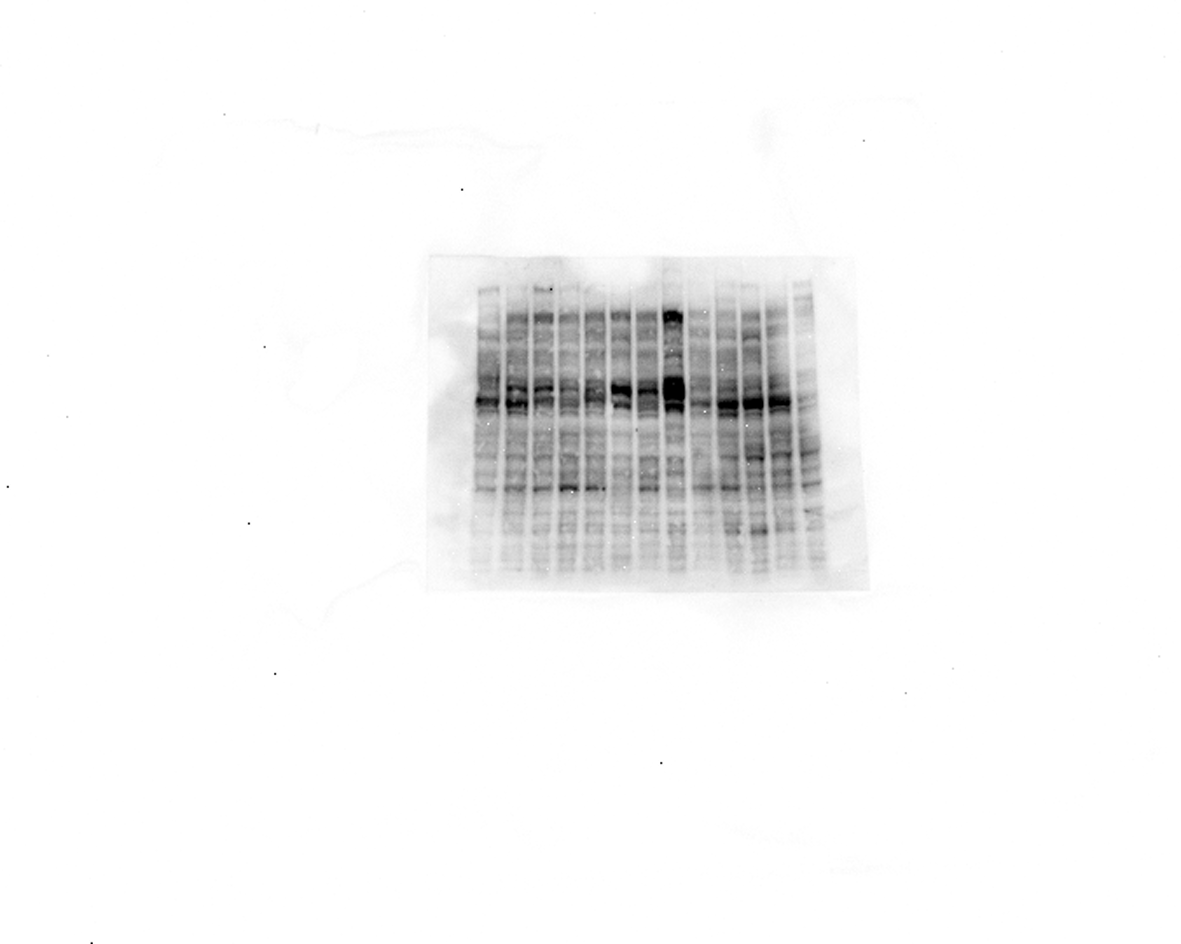

Supplement: Supplementary file 1 [file cancers-13-03404-s001.zip › CHEMI_03022020_185549.tif]

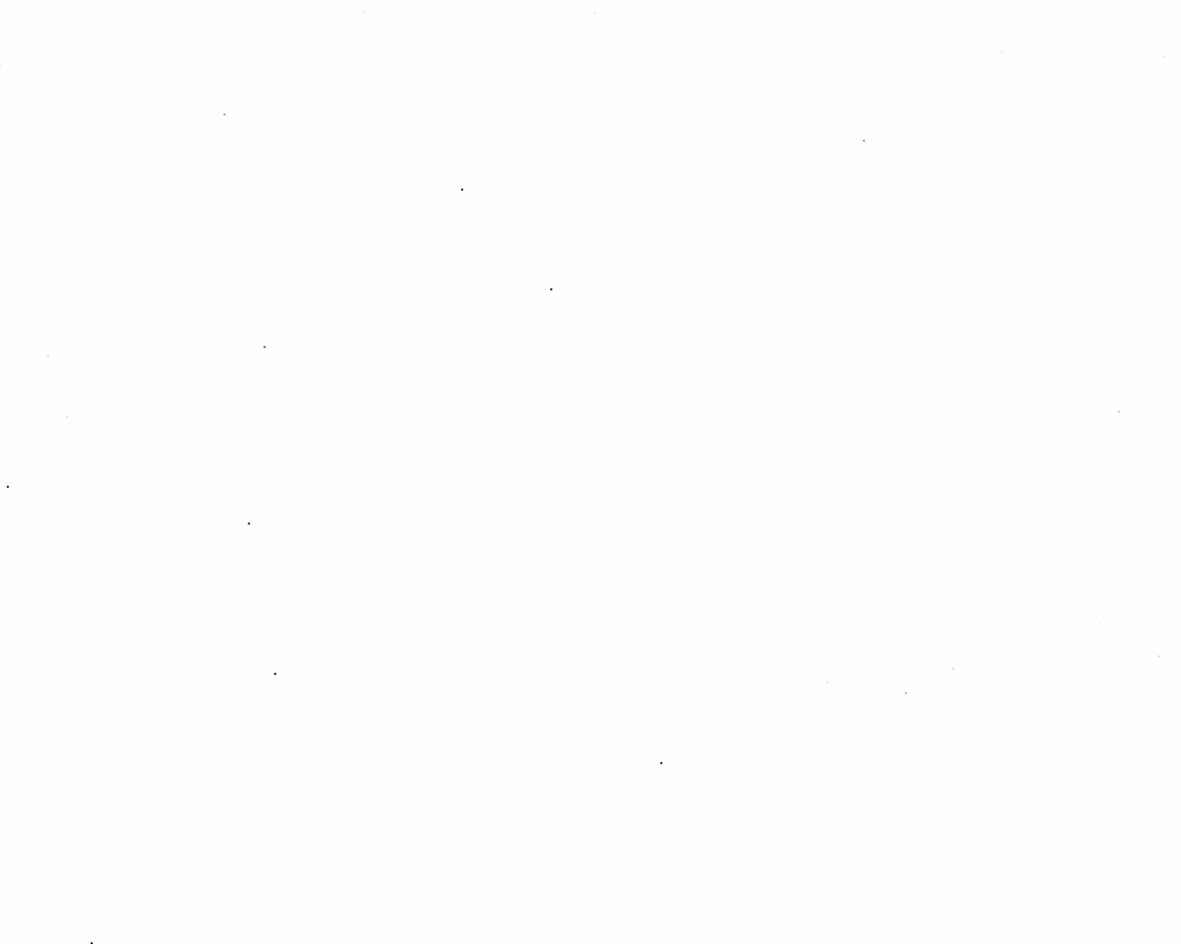

Supplement: Supplementary file 1 [file cancers-13-03404-s001.zip › CHEMI_03032020_125618.jpg]

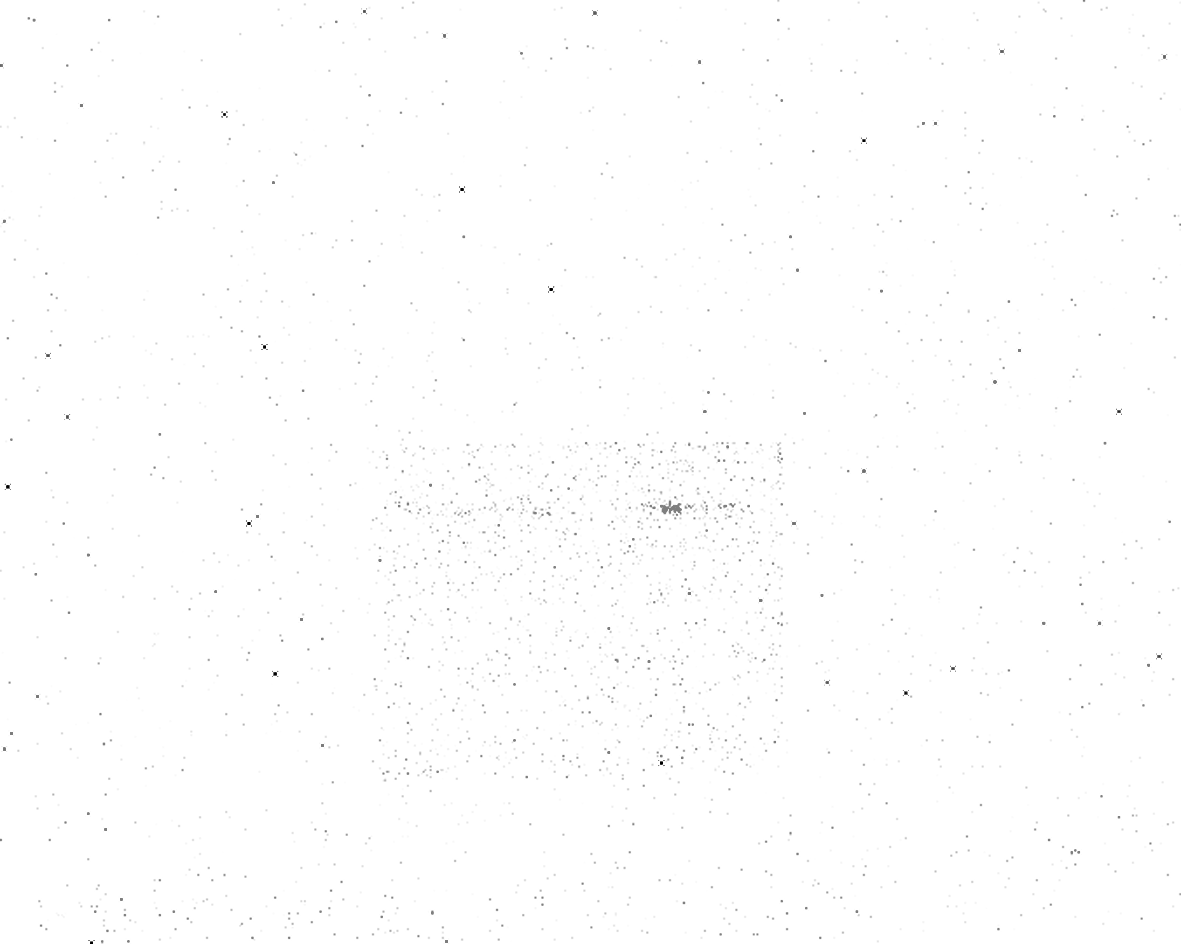

Supplement: Supplementary file 1 [file cancers-13-03404-s001.zip › CHEMI_03032020_125618.tif]

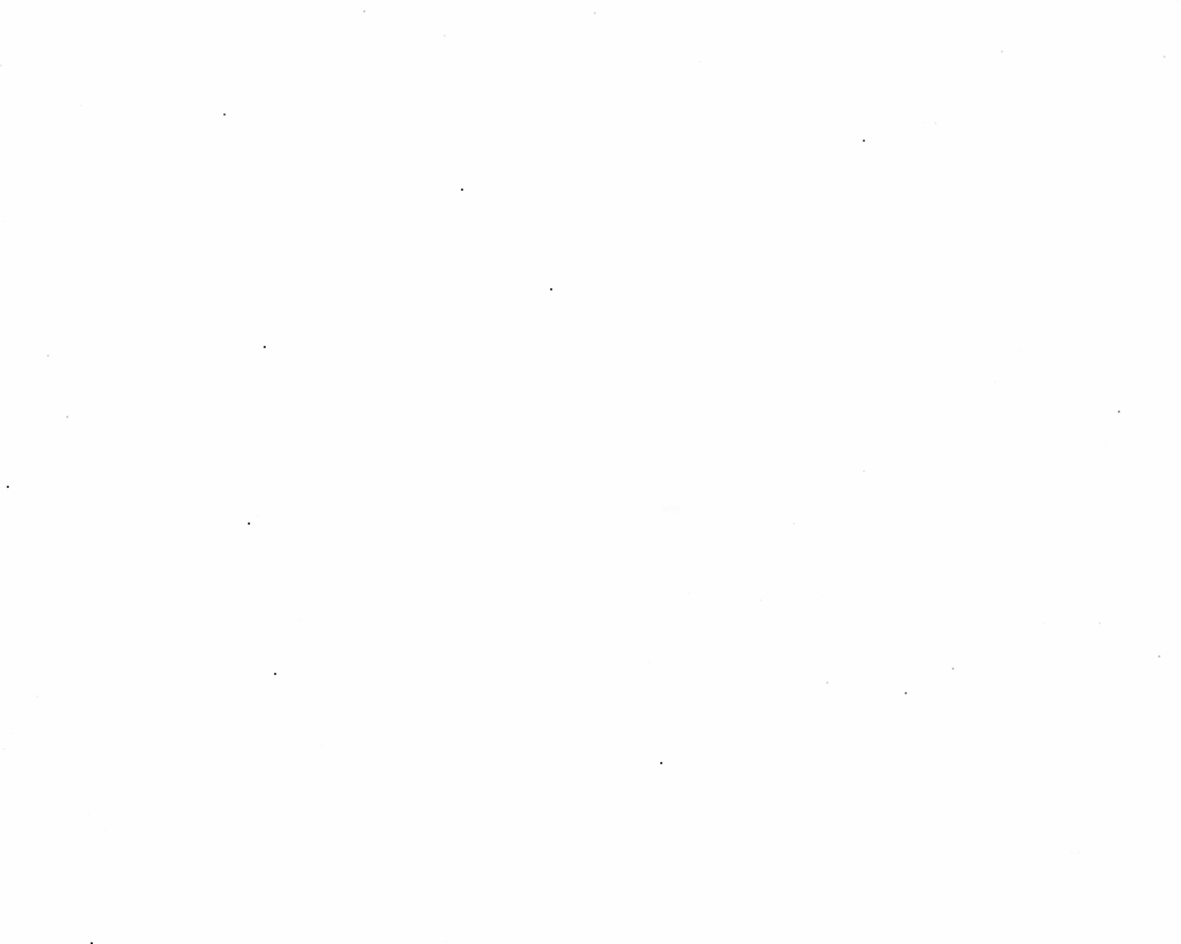

Supplement: Supplementary file 1 [file cancers-13-03404-s001.zip › CHEMI_03032020_125657.jpg]

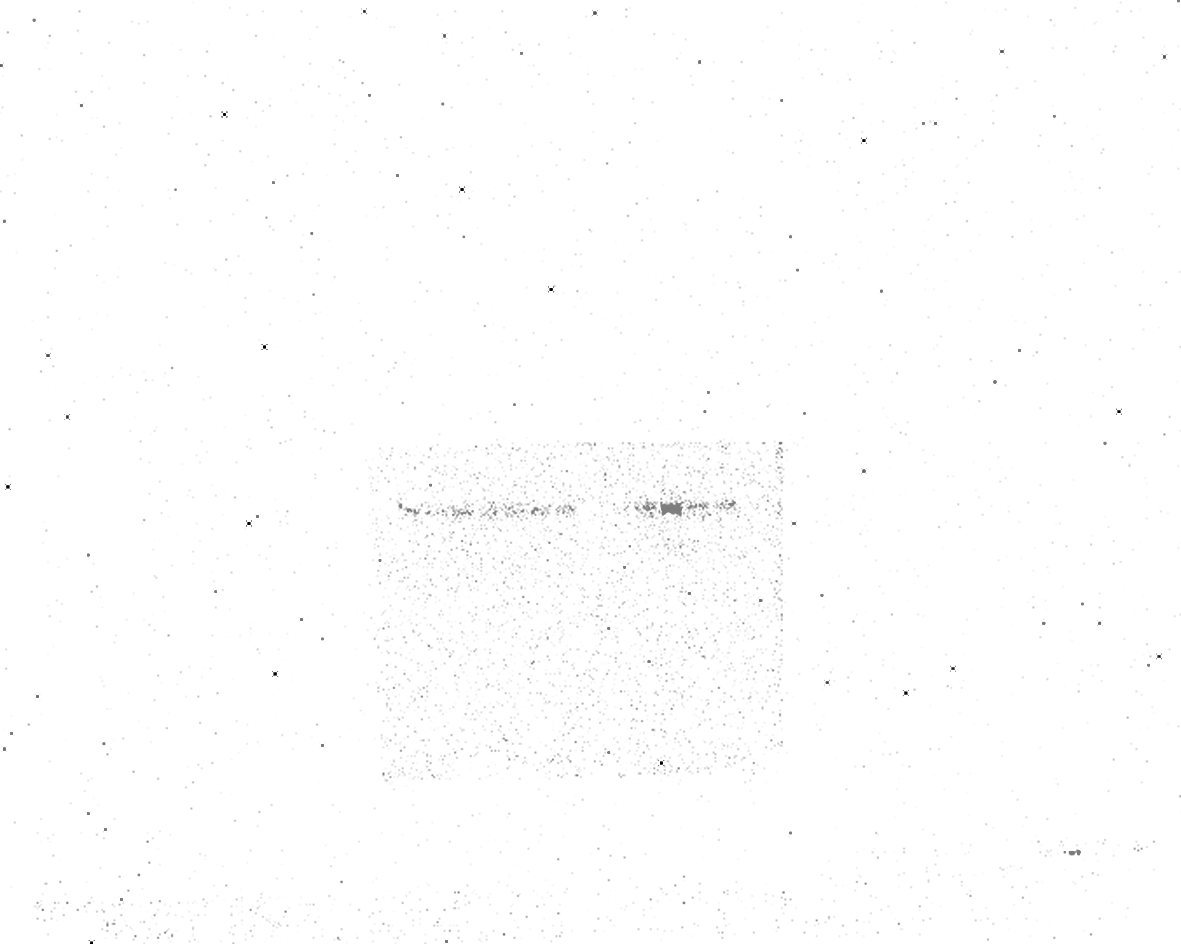

Supplement: Supplementary file 1 [file cancers-13-03404-s001.zip › CHEMI_03032020_125657.tif]

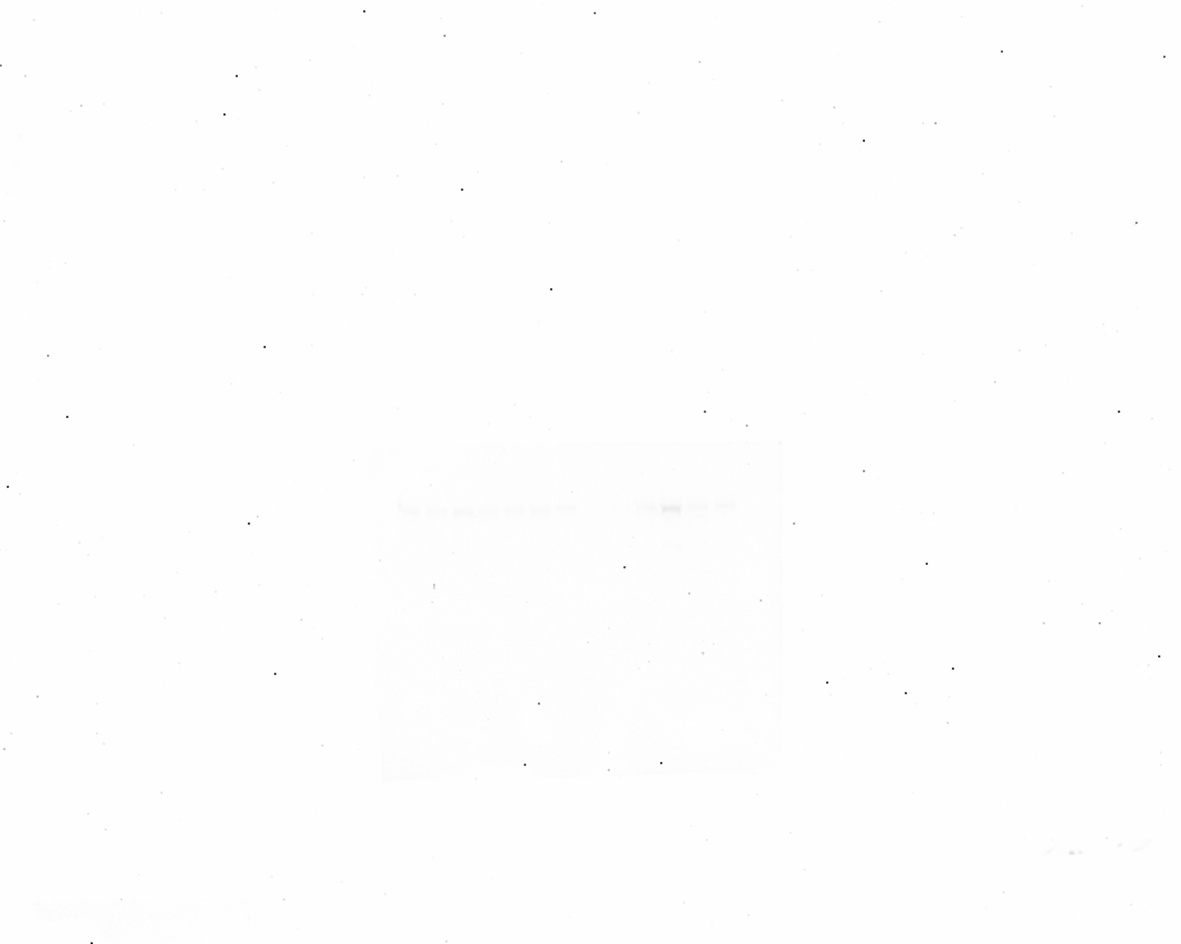

Supplement: Supplementary file 1 [file cancers-13-03404-s001.zip › CHEMI_03032020_125807.jpg]

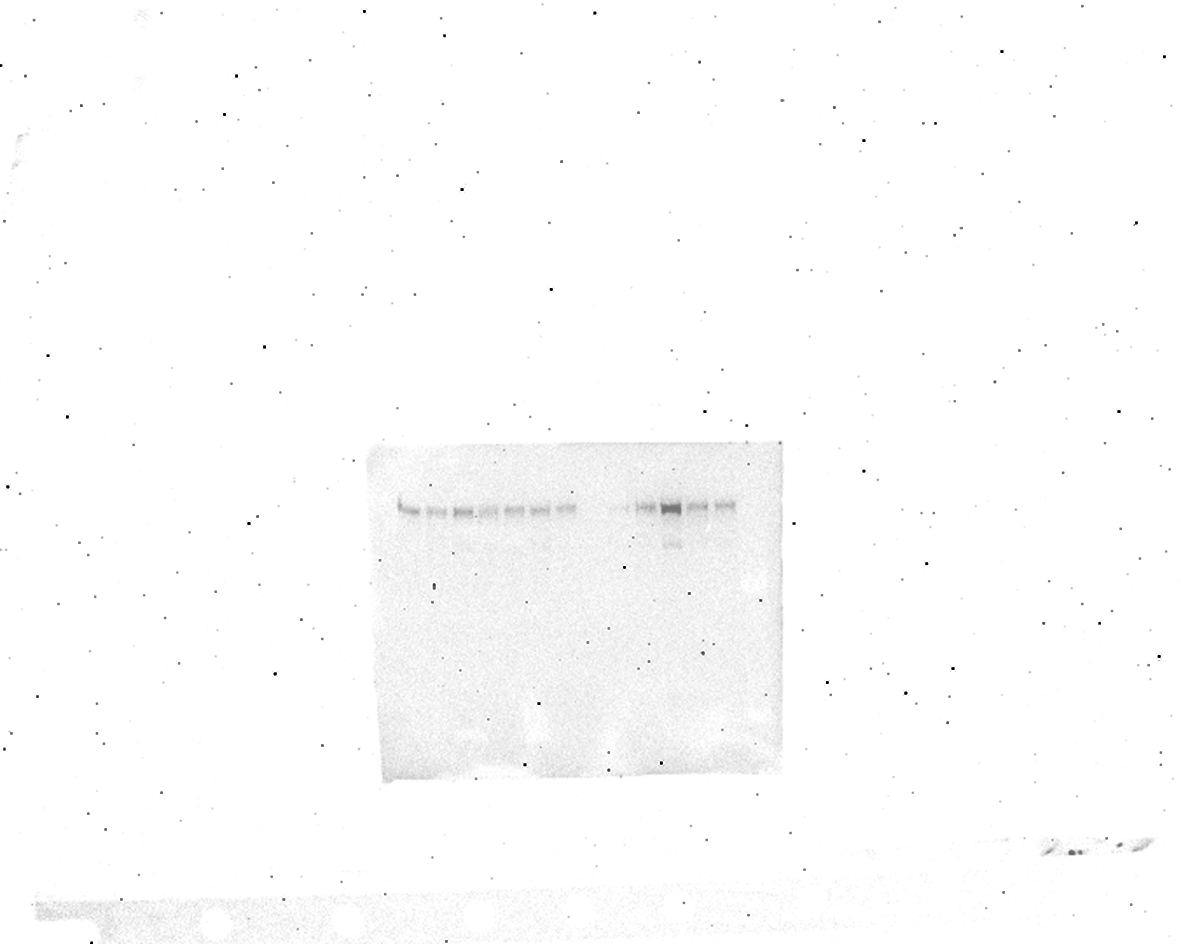

Supplement: Supplementary file 1 [file cancers-13-03404-s001.zip › CHEMI_03032020_125807.tif]

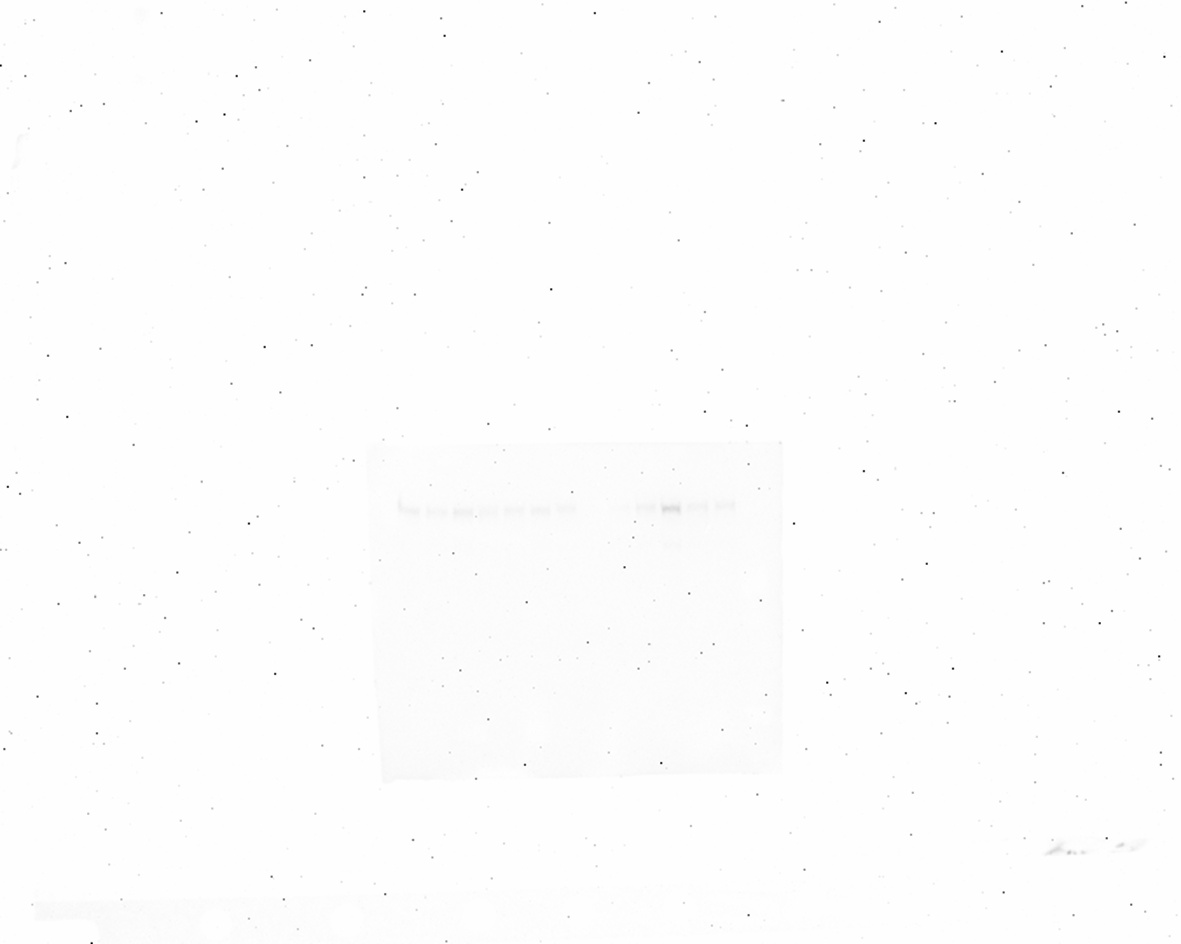

Supplement: Supplementary file 1 [file cancers-13-03404-s001.zip › CHEMI_03032020_130317.jpg]

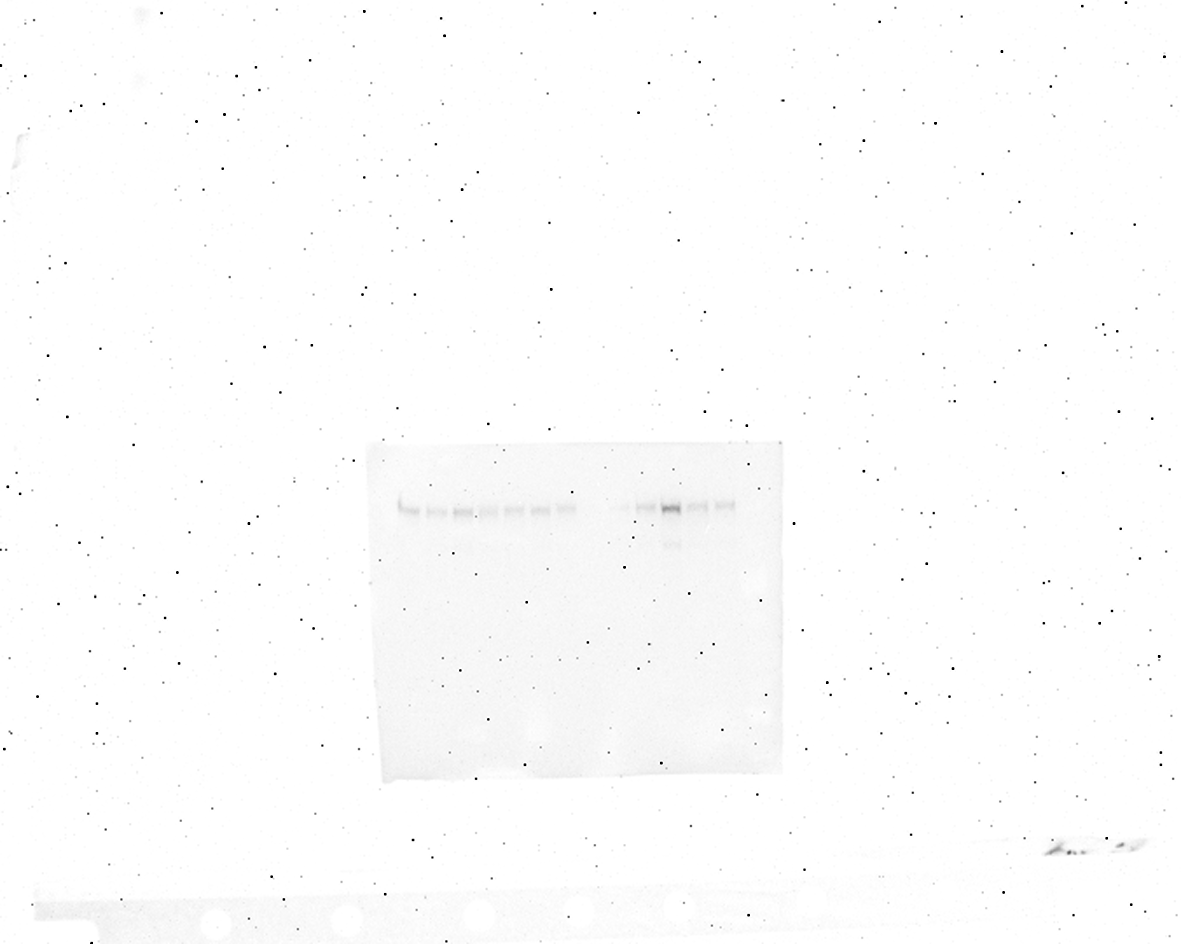

Supplement: Supplementary file 1 [file cancers-13-03404-s001.zip › CHEMI_03032020_130317.tif]

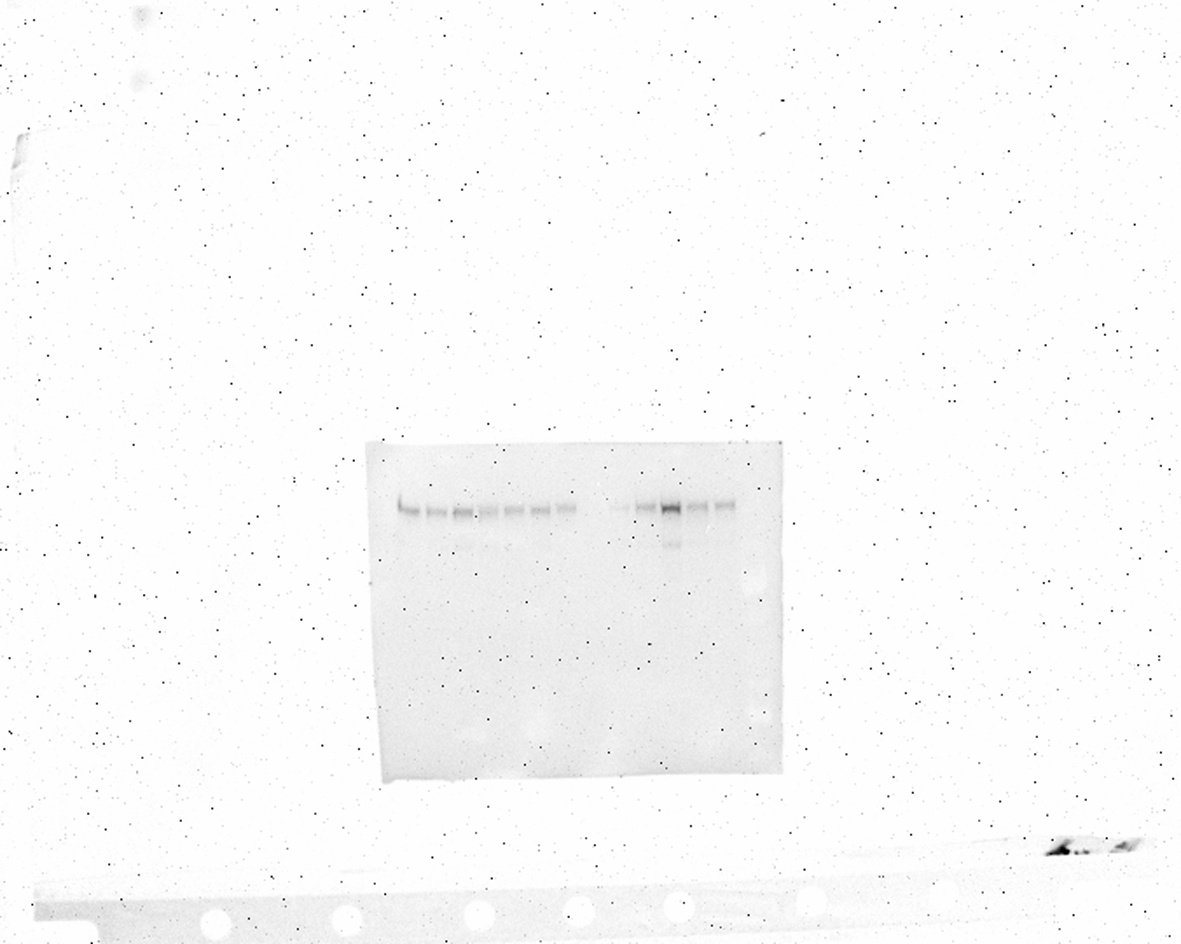

Supplement: Supplementary file 1 [file cancers-13-03404-s001.zip › CHEMI_03032020_131327.jpg]

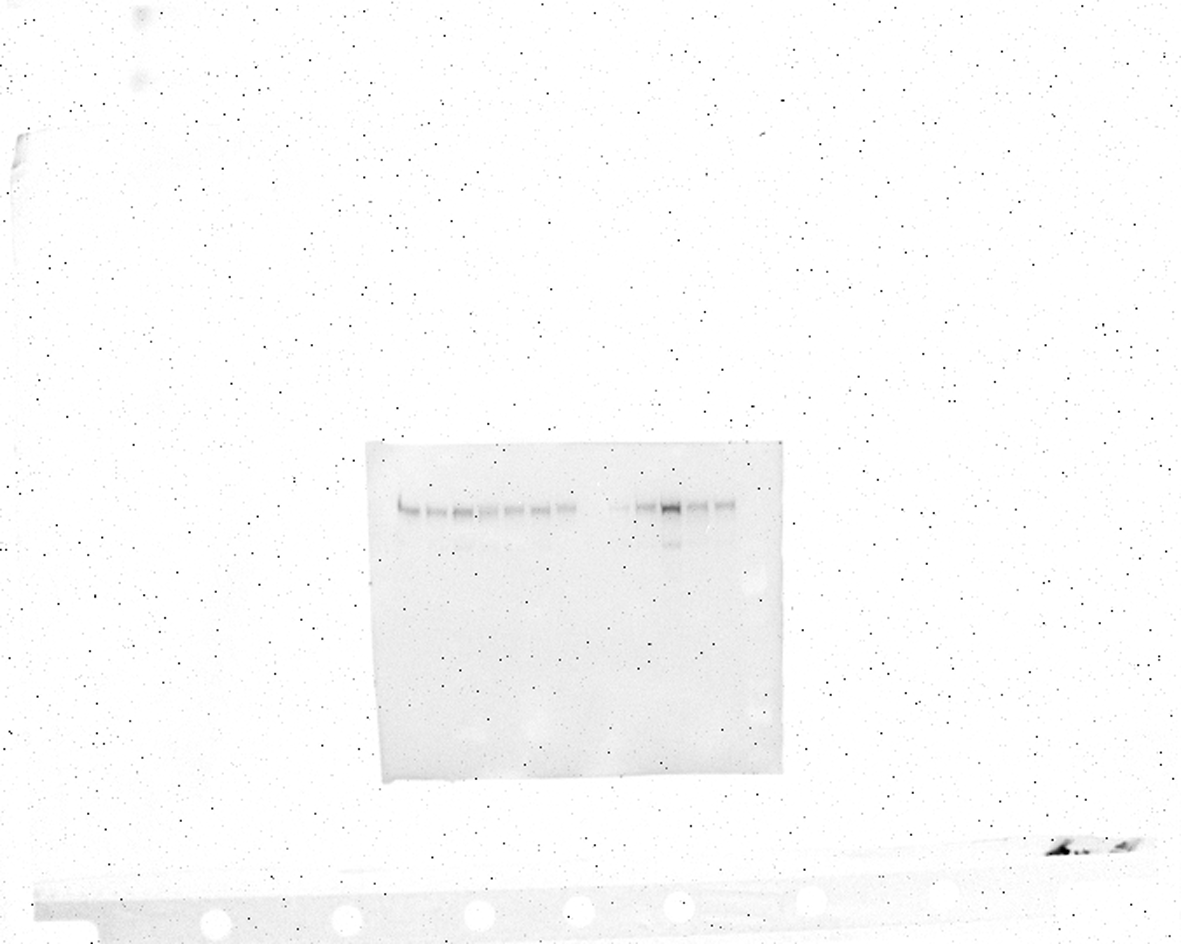

Supplement: Supplementary file 1 [file cancers-13-03404-s001.zip › CHEMI_03032020_131327.tif]

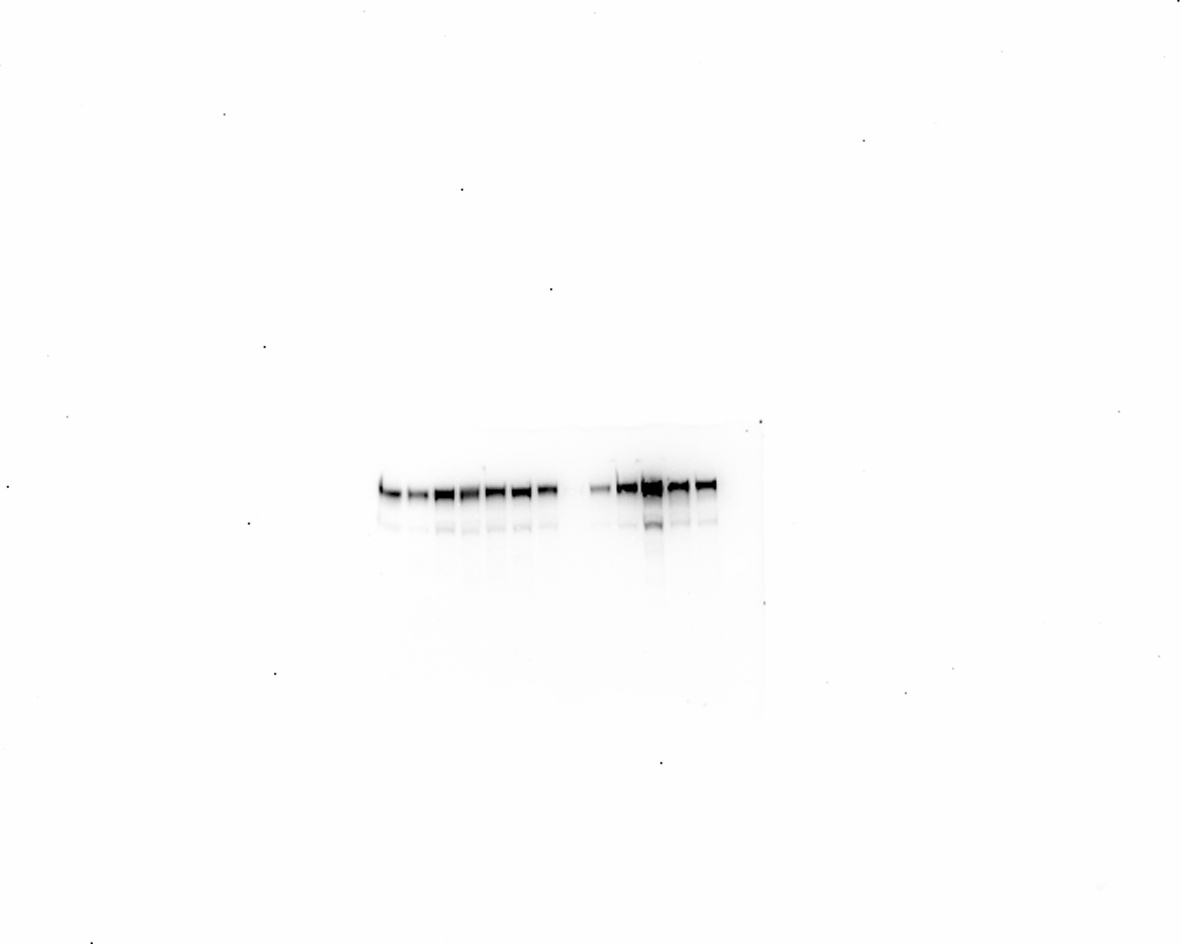

Supplement: Supplementary file 1 [file cancers-13-03404-s001.zip › CHEMI_03032020_133103.jpg]

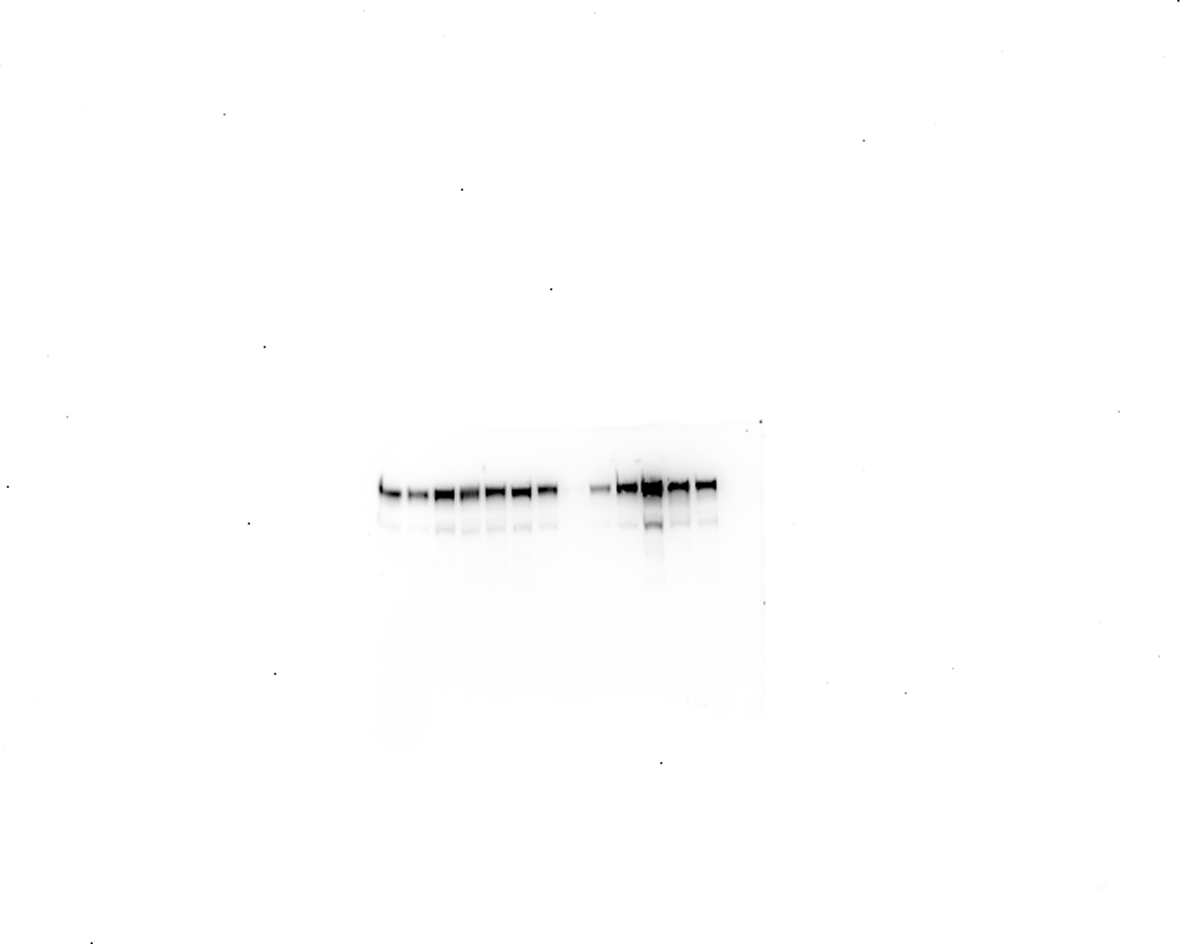

Supplement: Supplementary file 1 [file cancers-13-03404-s001.zip › CHEMI_03032020_133103.tif]

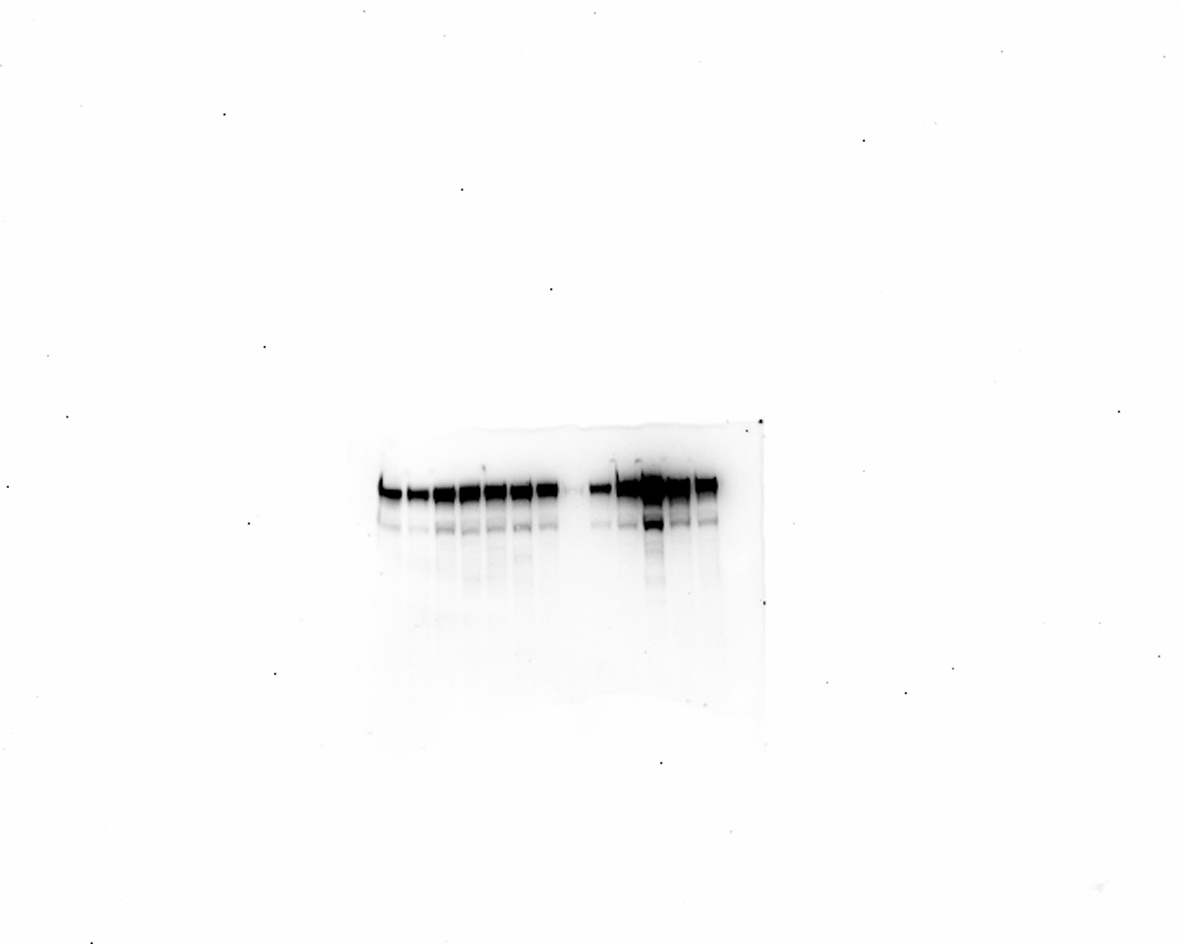

Supplement: Supplementary file 1 [file cancers-13-03404-s001.zip › CHEMI_03032020_133142.jpg]

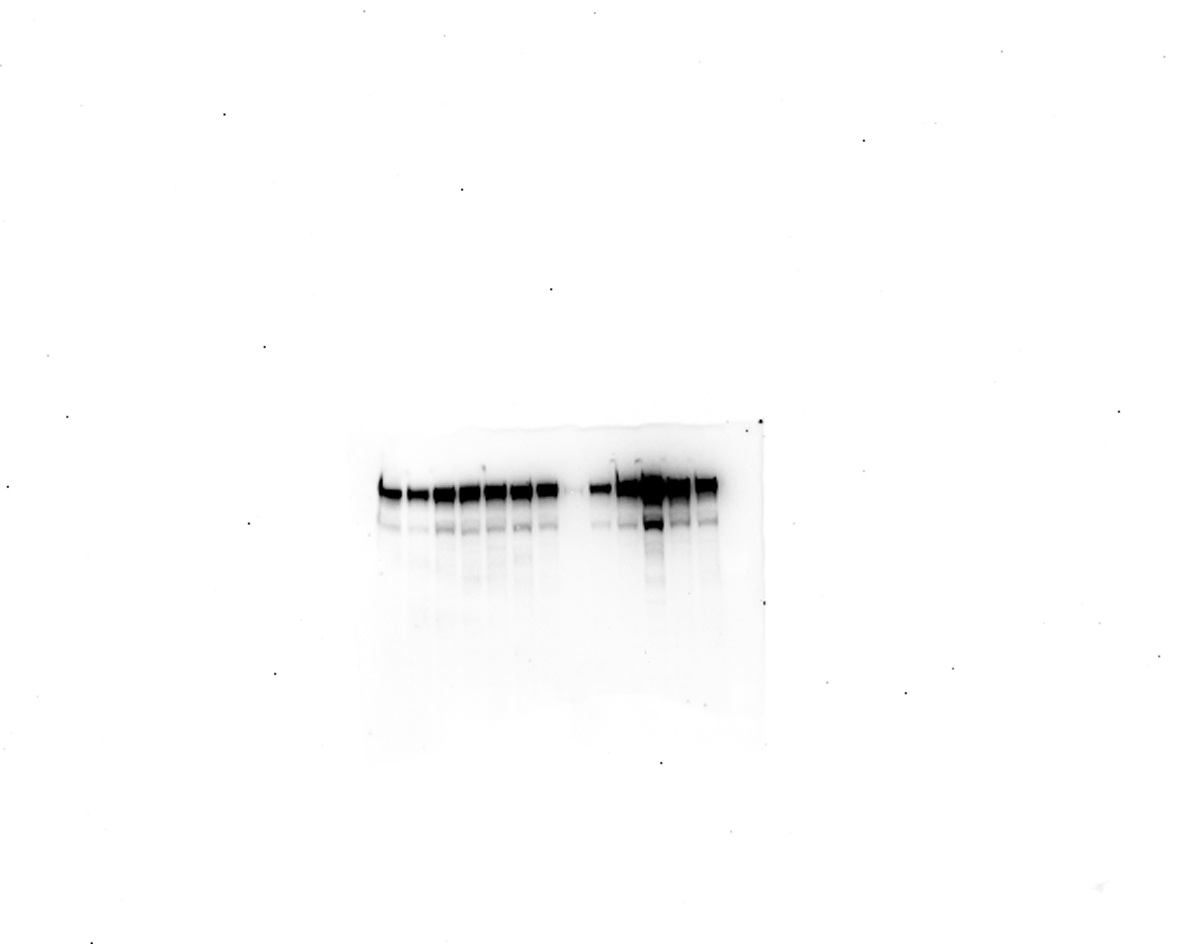

Supplement: Supplementary file 1 [file cancers-13-03404-s001.zip › CHEMI_03032020_133142.tif]

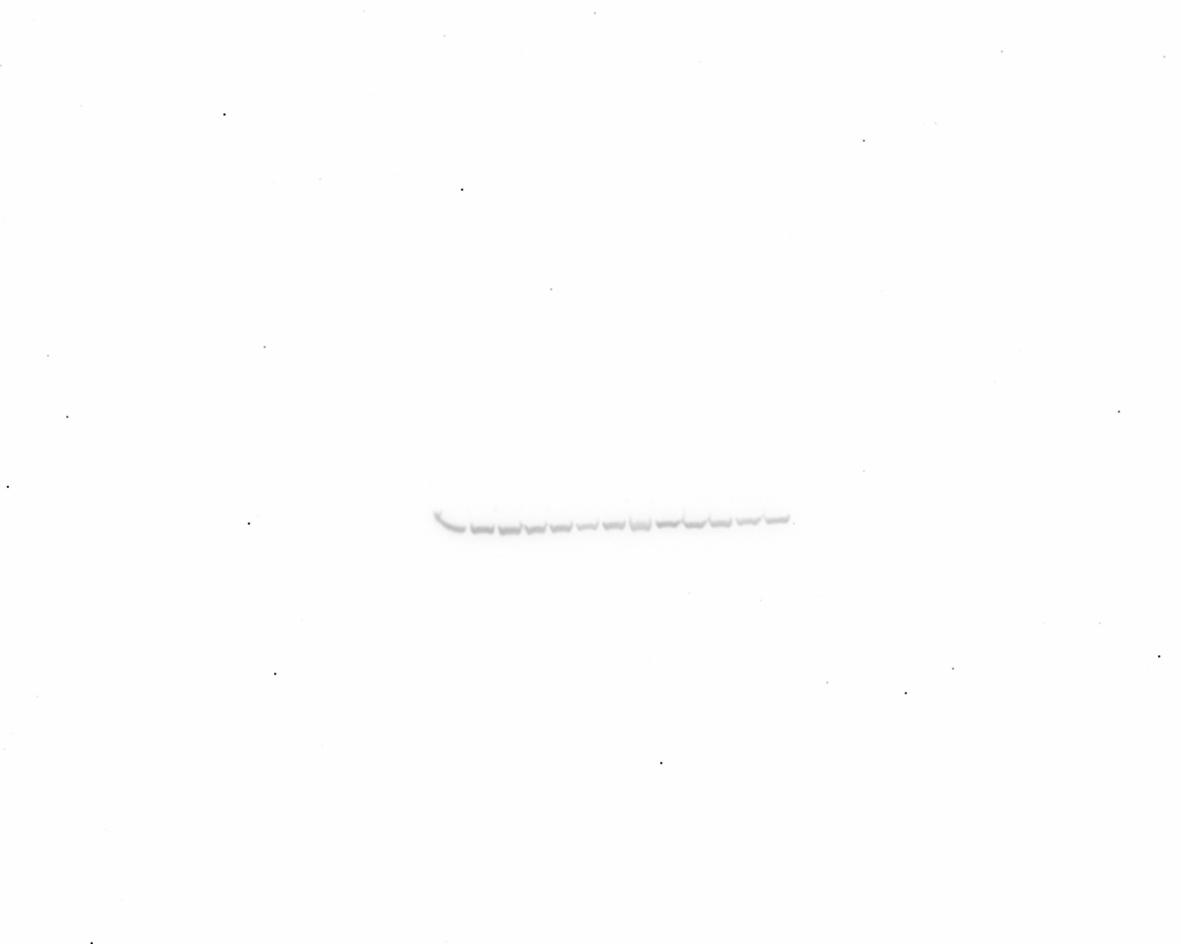

Supplement: Supplementary file 1 [file cancers-13-03404-s001.zip › CHEMI_03032020_151547.jpg]

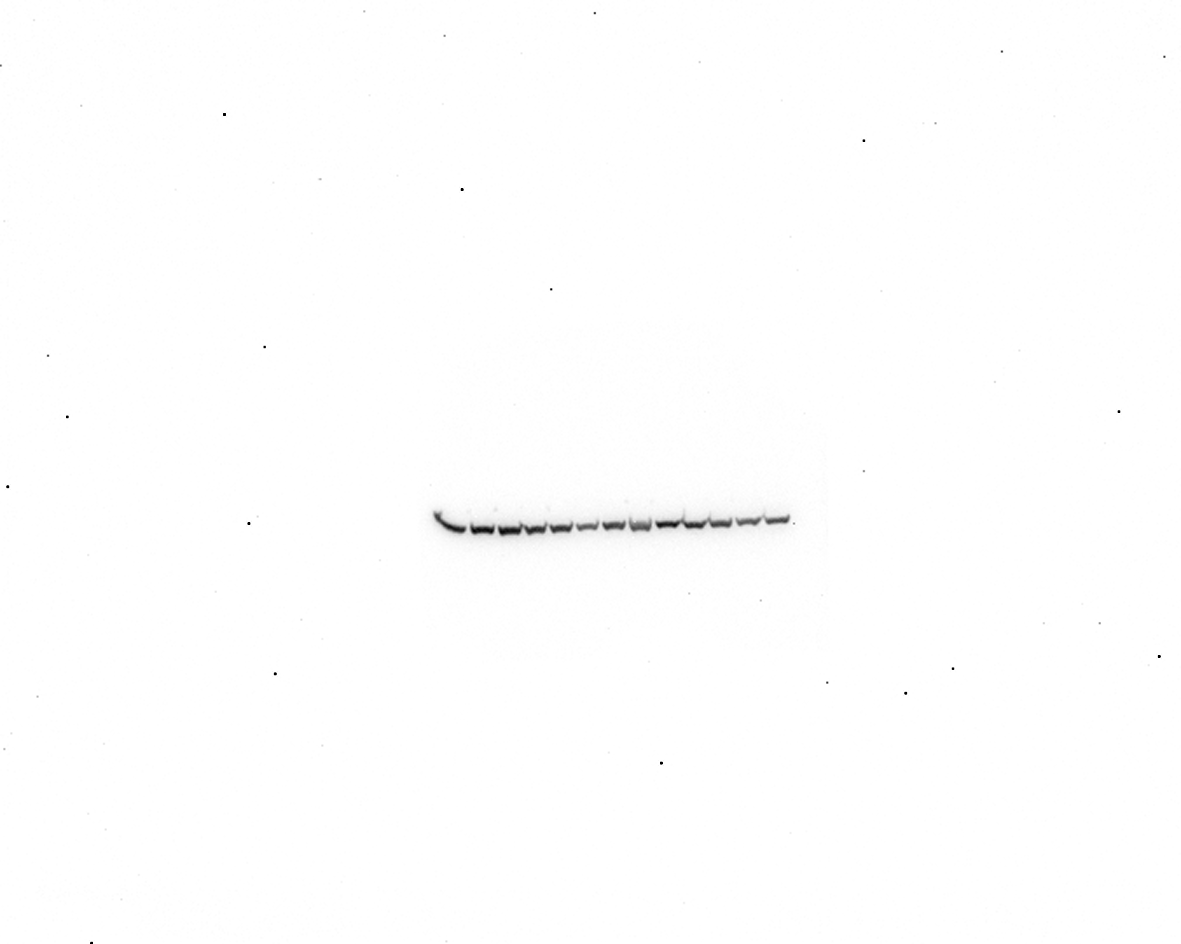

Supplement: Supplementary file 1 [file cancers-13-03404-s001.zip › CHEMI_03032020_151547.tif]

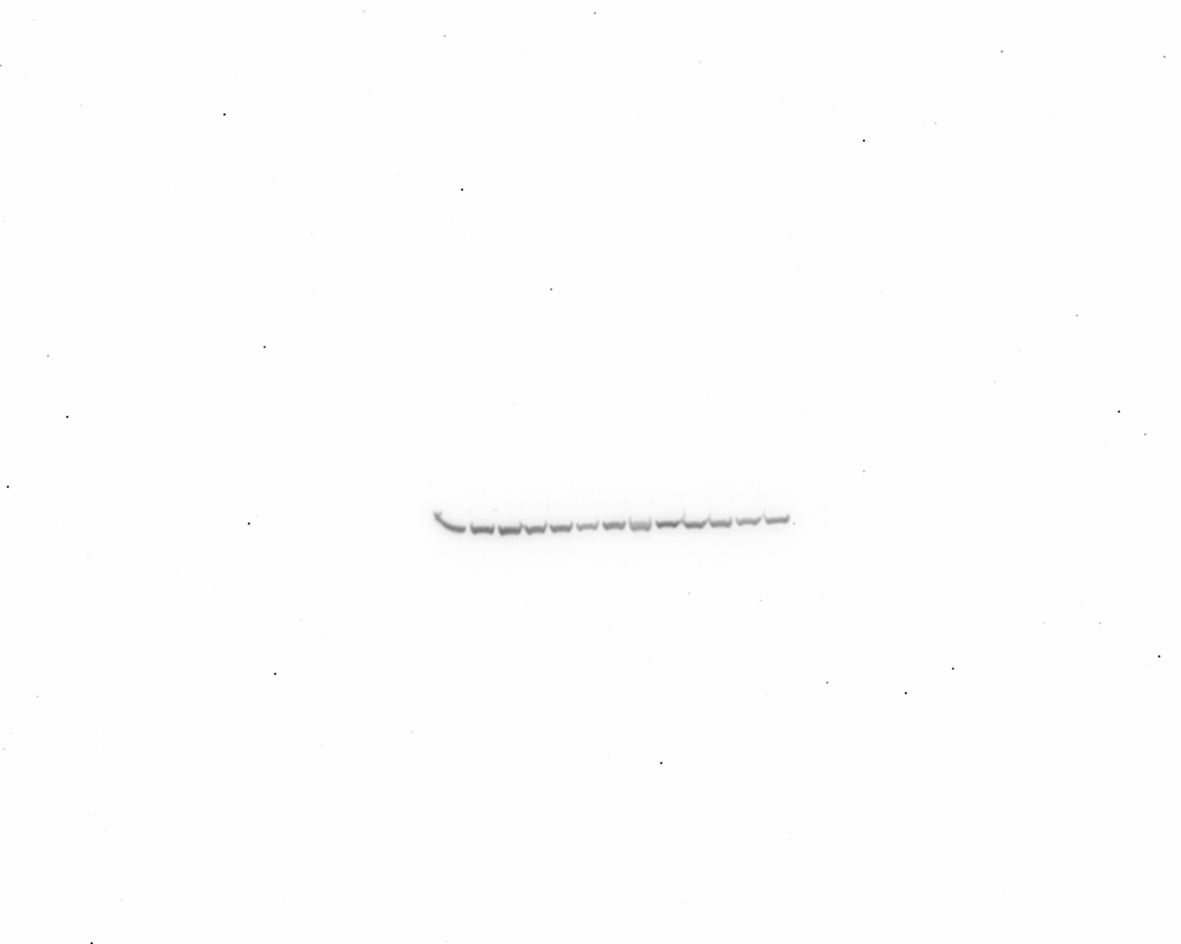

Supplement: Supplementary file 1 [file cancers-13-03404-s001.zip › CHEMI_03032020_151625.jpg]

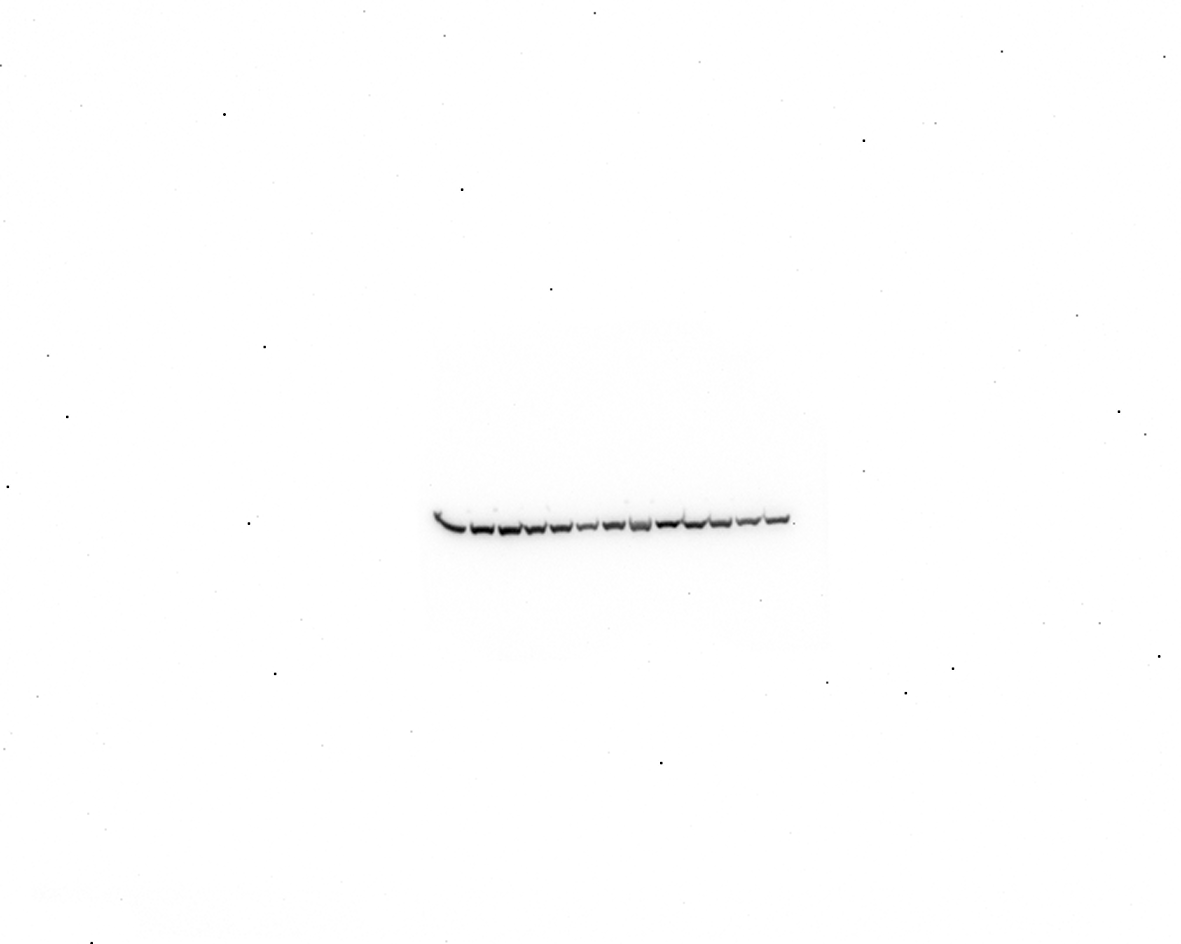

Supplement: Supplementary file 1 [file cancers-13-03404-s001.zip › CHEMI_03032020_151625.tif]

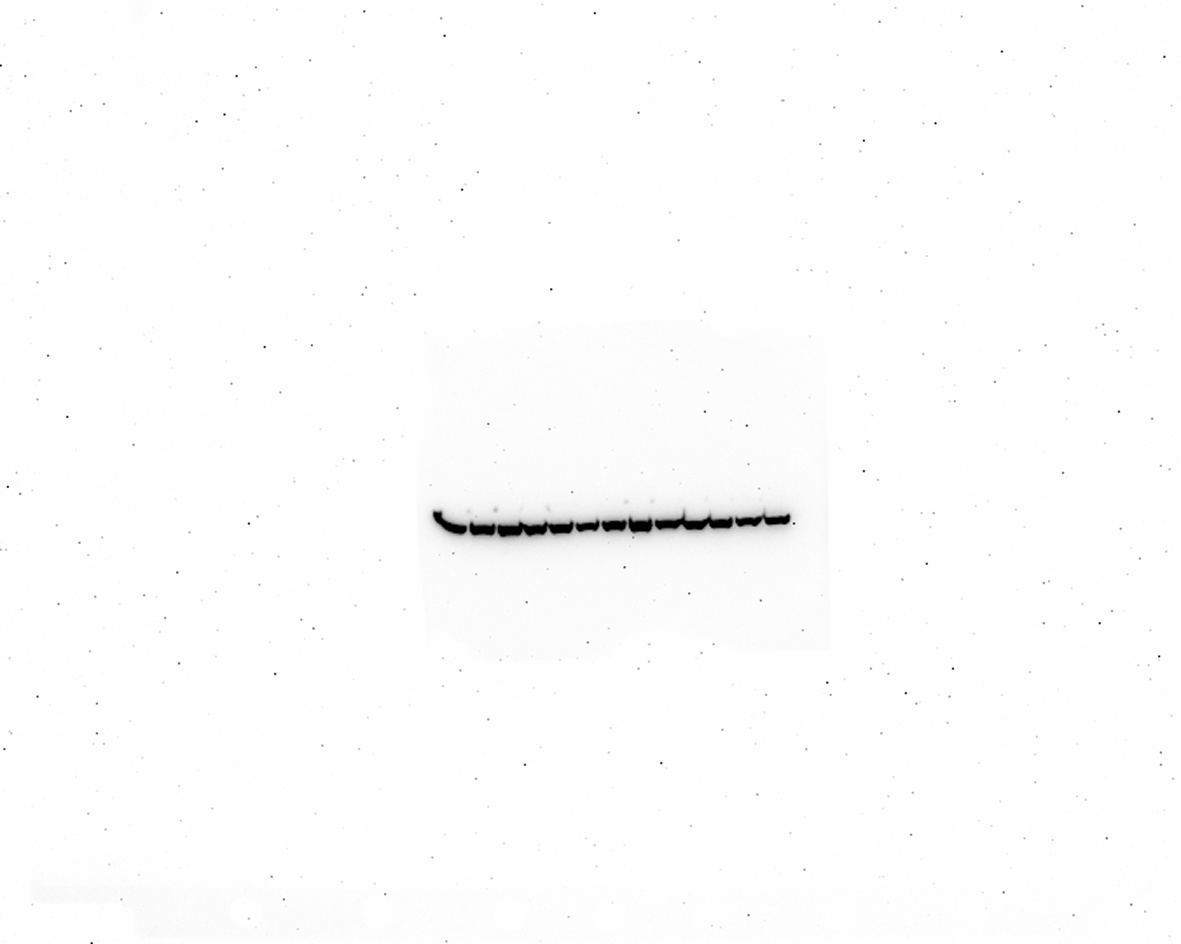

Supplement: Supplementary file 1 [file cancers-13-03404-s001.zip › CHEMI_03032020_151735.jpg]

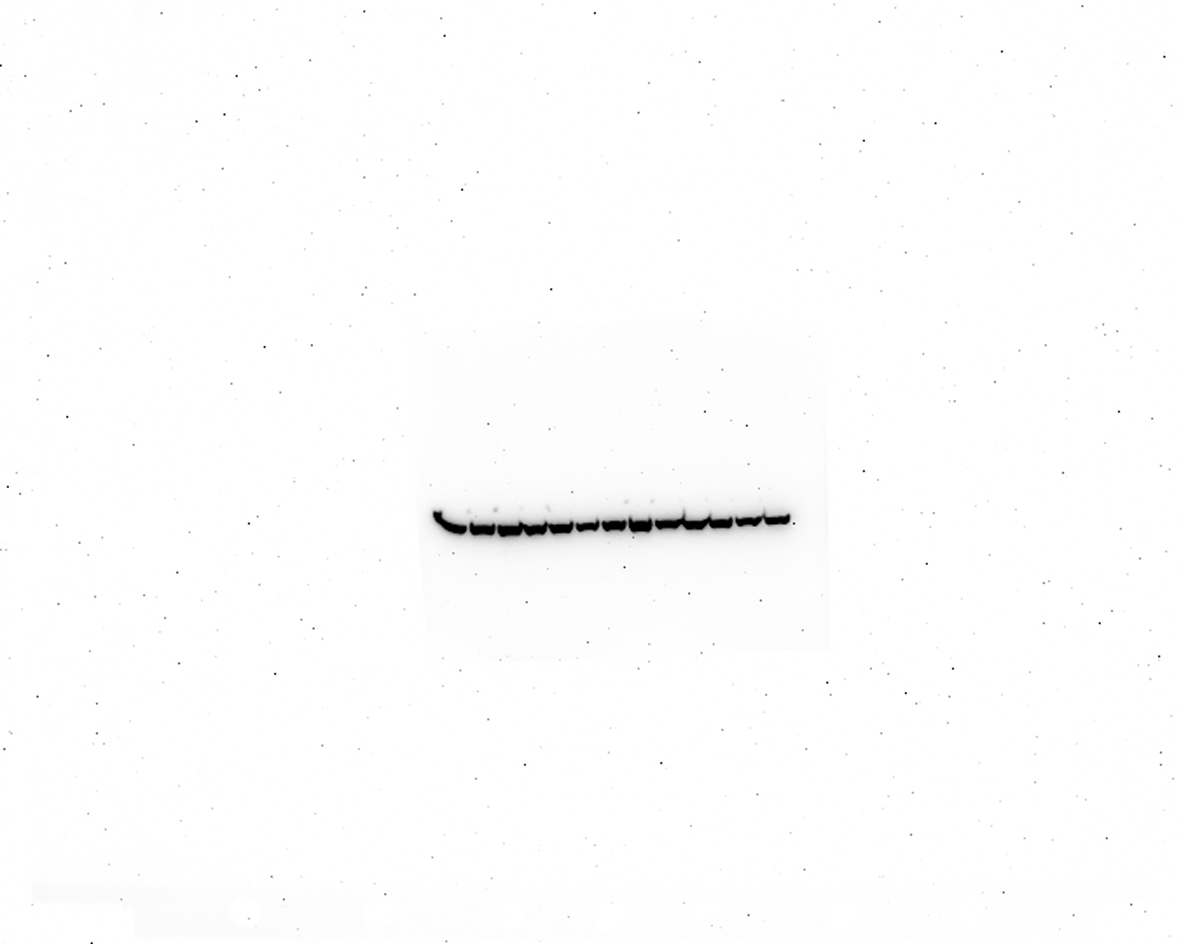

Supplement: Supplementary file 1 [file cancers-13-03404-s001.zip › CHEMI_03032020_151735.tif]
